# Supplementary material for: Metabolic reprogramming via LDHB confers resistance to lactate-induced dysfunction and enhanced antitumor activity in hiPSC-Derived NK cells
Source: Stem Cell Reports. 2026 Aug 13;21(9):103048. doi: 10.1016/j.stemcr.2026.103048 (PMC13555463; doi:10.1016/j.stemcr.2026.103048)
Supplement: Supplementary file 1 [file mmc1.pdf]

Stem Cell Reports, Volume *21*

Supplemental Information

Metabolic reprogramming via LDHB confers resistance to lactate-induced dysfunction and enhanced antitumor activity in hiPSC-Derived NK cells

Zhishuai Zhang, Yuchan Mai, Jun Tang, Jiaying Ning, Yanjiao Qin, Jiaming Gu, Zhaozong Cao, Jian Liu, Tiancheng Zhou, Junwei Wang, Jeane L. Pan, Jiaheng Chen, Yanling Zhu, and Guangjin Pan

supplemental 1

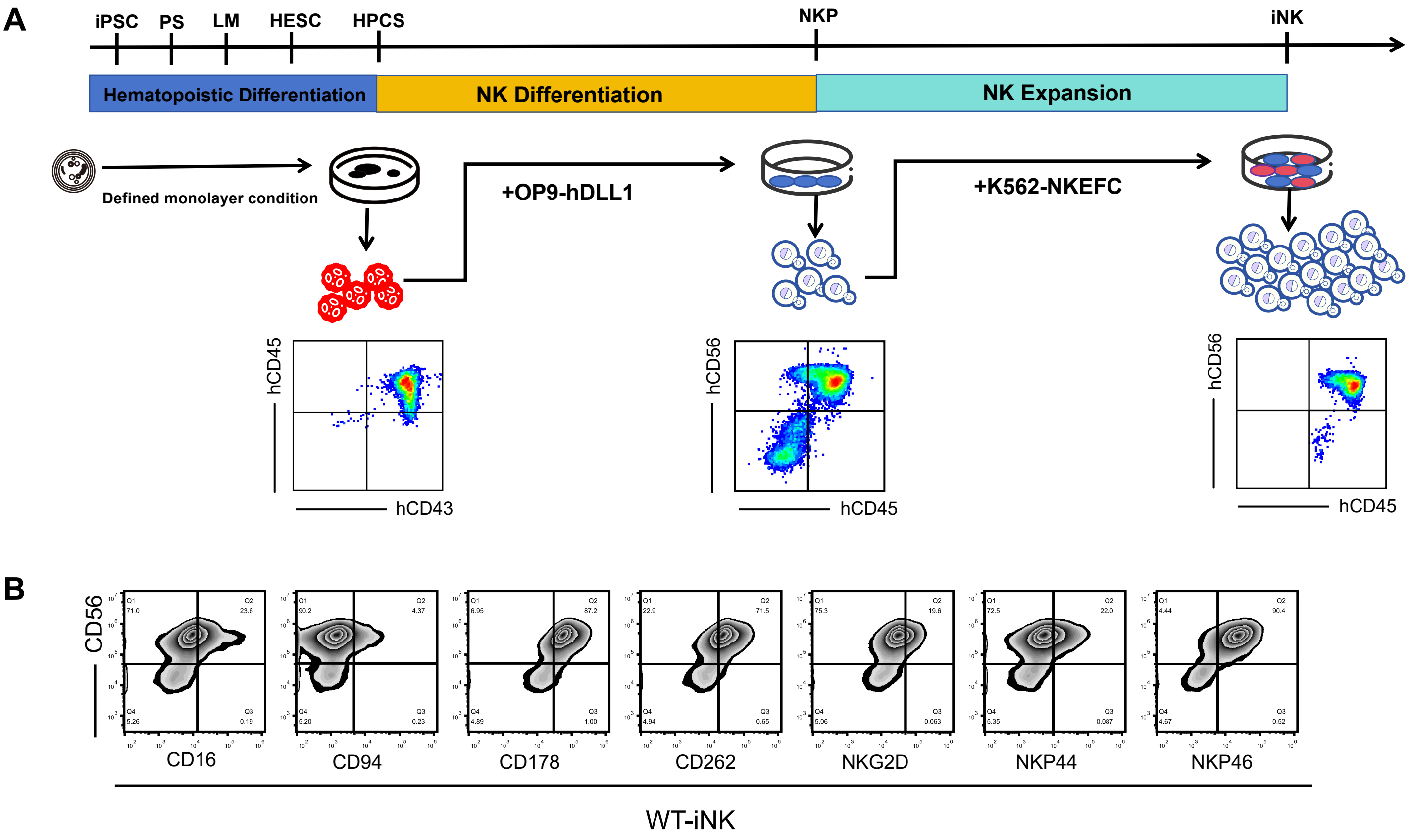

supplemental 2

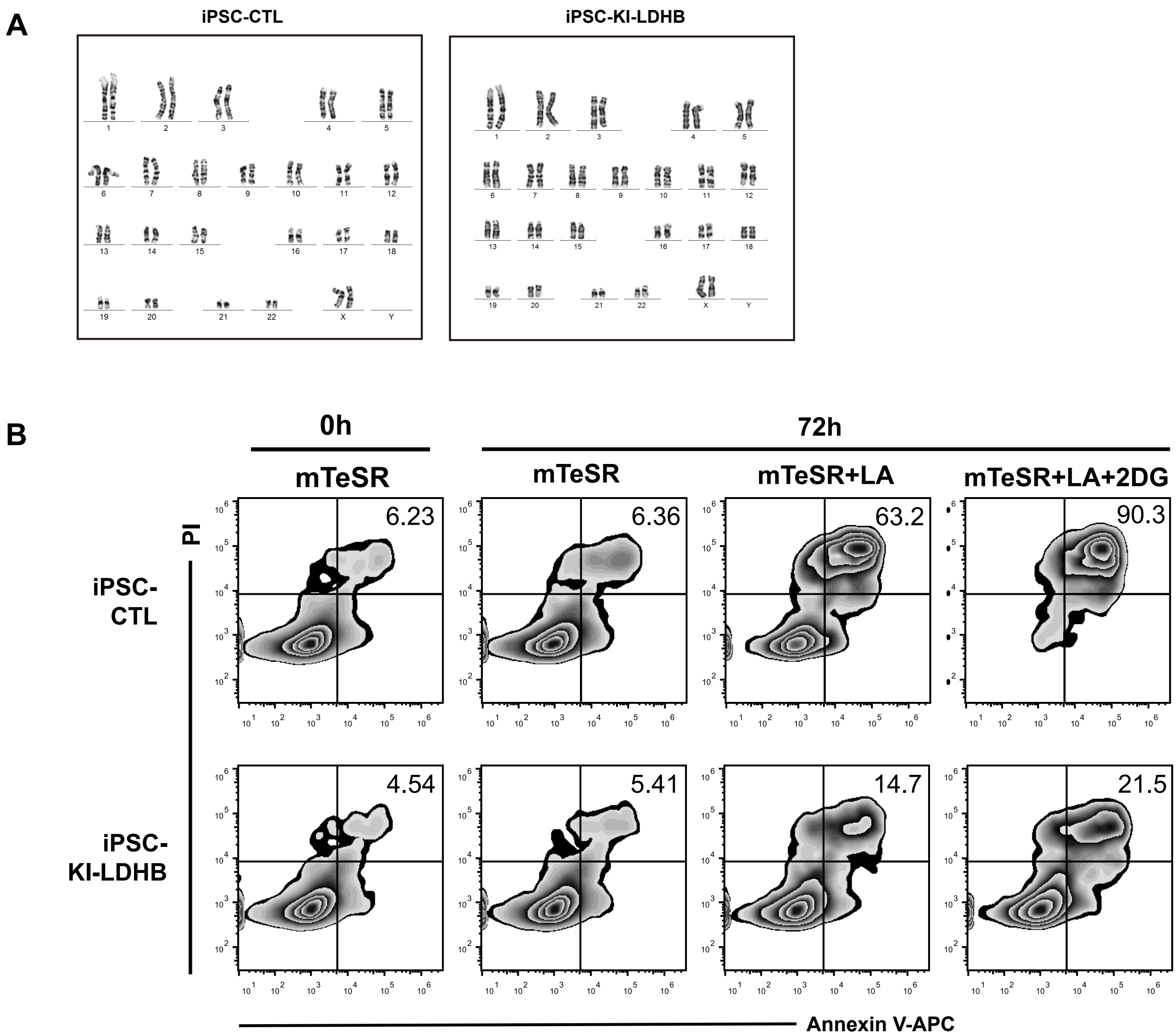

supplemental 3

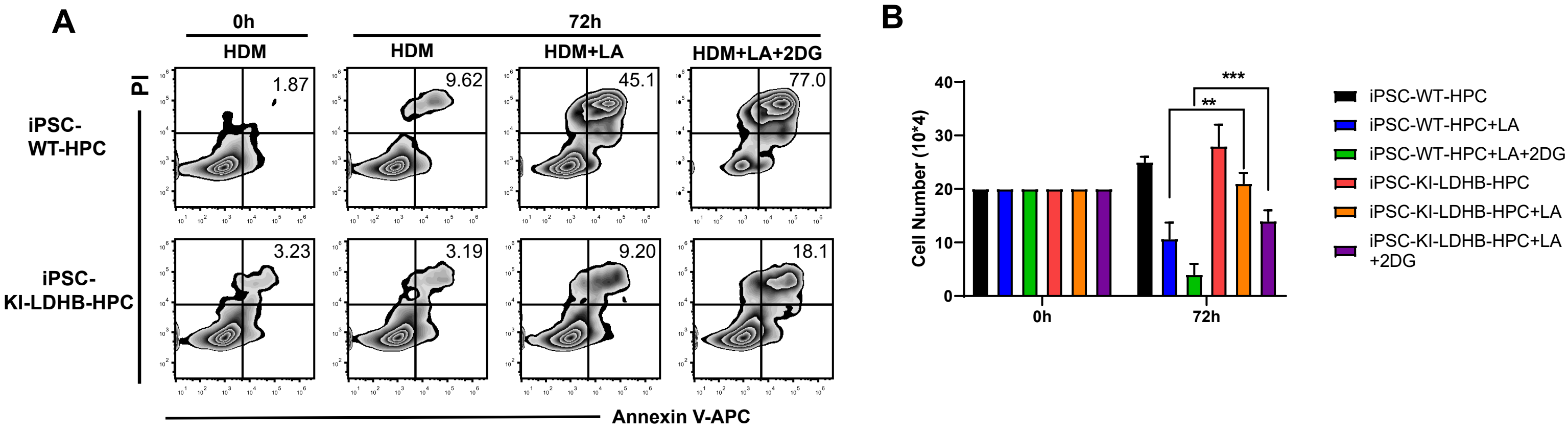

supplemental 4

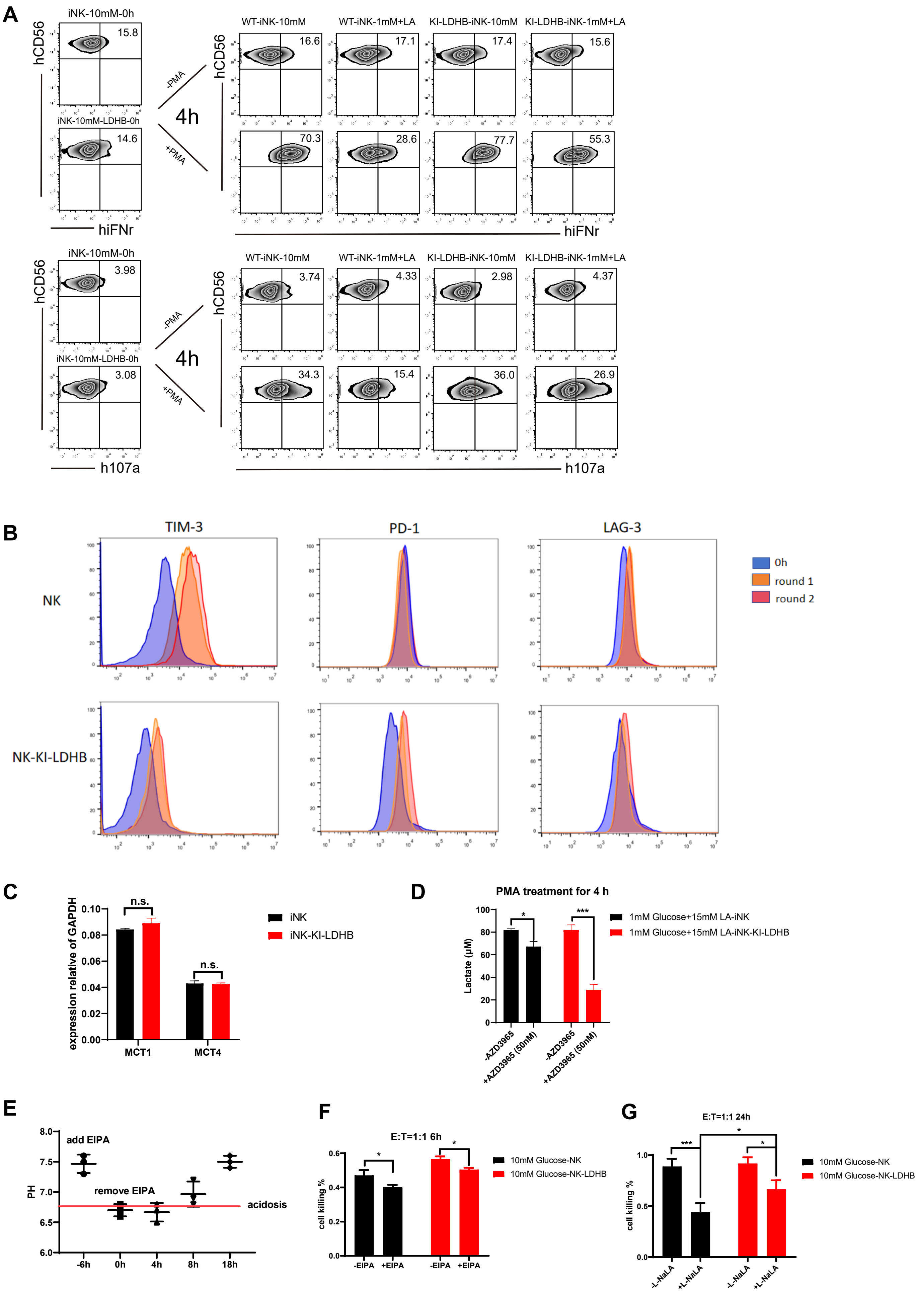

Supplemental 5

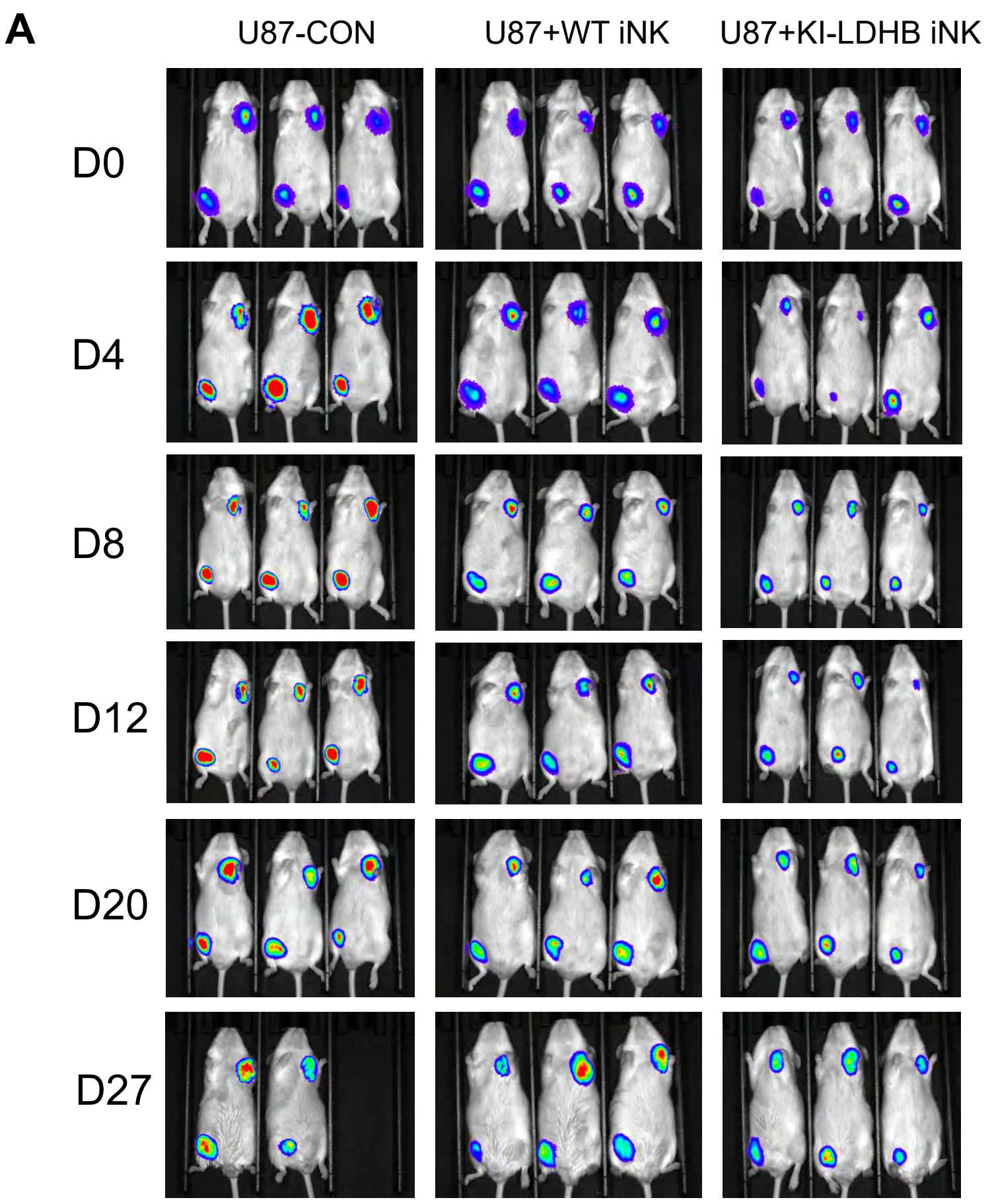

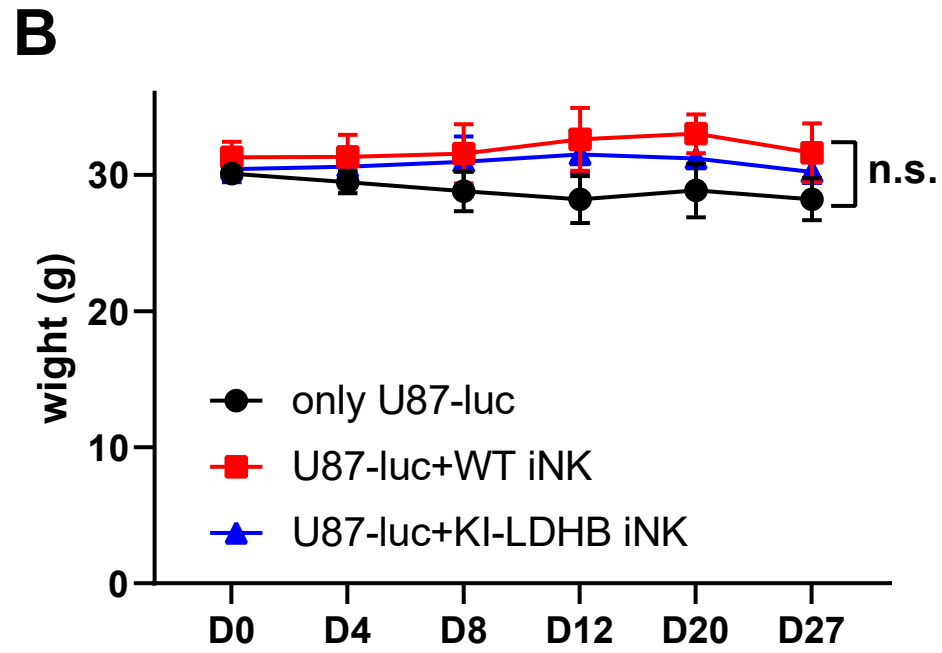

Supplemental Figure 1.

(A) Schematic of the differentiation protocol from hiPSCs to hematopoietic progenitor cells (iHPCs) to iNKs.

(B) Flow cytometry analysis characteristic NK cell surface markers on derived iNKs.

Supplemental Figure 2.

(A) Karyotype analysis of LDHB-knock-in iPSC clones.

(B) Flow cytometry analysis of apoptosis in WT and LDHB-hiPSCs after 72-hour culture in mTeSR, or mTeSR + lactate, or mTeSR + lactate + 2-DG.

Supplemental Figure 3.

(A) Flow cytometry analysis of apoptosis in WT and LDHB_iHSPCs after 72-hour culture in differentiation medium or medium + lactate, or medium + lactate + 2-DG.

(B) Quantification of cell numbers for the experiment in (A). *N=3*, Data are from three independent differentiation batches.

Supplemental Figure 4.

(A) Flow cytometry analysis of IFN-γ and Granzyme B secretion in WT and LDHB-iNKs, with or without lactate exposure, following PMA/ionomycin stimulation.

(B) Flow cytometry was used to detect the expression of exhaustion-related genes in iNK and iNK-KI-LDHB cells during multiple rounds of killing.

(C) Lactate concentration in iNK cells cultured in 1 mM glucose + 15 mM L-lactate medium , following 4 h PMA treatment. AZD3965 is a specific inhibitor of MCT-1, which can inhibit lactate influx.*N=3*, Data are from three independent differentiation batches. *$p < 0.05$, ***$p < 0.001$.

(D) Relative mRNA expression levels of MCT1 and MCT4 in iNK and iNK-KI-LDHB, normalized to GAPDH. *N=3*, Data are from three independent differentiation batches.

(E) Cells were treated with EIPA 40μM for 6 hours prior to the start of the experiment (time -6h). Upon removal of EIPA (time 0h), the intracellular pH gradually recovered but remained below the acidosis threshold (indicated by the red dashed line) throughout the observation period. *N=3*, Data are from three independent differentiation batches. Data are presented as mean ± SEM.

(F) Cytotoxic activity of iNK and iNK-KI-LDHB cells against U87 target cells at an effector-to-target (E:T) ratio of 1:1, measured after 6 hours of co-culture in EIPA medium. *N=3*, Data are from three independent differentiation batches. Data are mean ± SD . $*p < 0.05$, $**p < 0.01$.

(G) Cytotoxic activity of iNK and iNK-KI-LDHB cells against U87 target cells at an effector-to-target (E:T) ratio of 1:1, measured after 24 hours of co-culture in the presence or absence of 15 mM sodium L-lactate (L-A). *N=3*, Data are from three independent differentiation batches. Data are mean ± SD. $*p < 0.05$, $**p < 0.01$.

Supplemental Figure 5.

(A) Representative in vivo bioluminescence images of mice bearing U87-luc tumors treated with iNK or iNK-KI-LDHB.

(B) Body weight measurements of mice throughout the treatment period. *N=3*, with each set of data sourced from different mice.
