# Supplementary material for: Metabolic reprogramming via LDHB confers resistance to lactate-induced dysfunction and enhanced antitumor activity in hiPSC-Derived NK cells
Source: Stem Cell Reports. 2026 Aug 13;21(9):103048. doi: 10.1016/j.stemcr.2026.103048 (PMC13555463; doi:10.1016/j.stemcr.2026.103048)
Supplement: Supplementary file 2 [file mmc2.pdf]

STEM CELL REPORTS
Article
OPEN ACCESS

Metabolic reprogramming via LDHB confers resistance to lactate-induced dysfunction and enhanced antitumor activity in hiPSC-Derived NK cells

Zhishuai Zhang,[1,2] Yuchan Mai,[1,3] Jun Tang,[4] Jiaying Ning,[4] Yanjiao Qin,[1,3] Jiaming Gu,[4] Zhaozong Cao,[1,5] Jian Liu,[1,3] Tiancheng Zhou,[1,3] Junwei Wang,[6] Jeane L. Pan,[7] Jiaheng Chen,[6] Yanling Zhu,[1,2,3,*] and Guangjin Pan[1,2,3,4,8,*]

[1]Key Laboratory of Immune Response and Immunotherapy, Guangzhou Institutes of Biomedicine and Health, Chinese Academy of Sciences, Guangzhou 510530, China
[2]University of Chinese Academy of Sciences, Beijing 100049, China
[3]Guangdong Provincial Key Laboratory of Stem Cell and Regenerative Medicine, Guangdong-Hong Kong Joint Laboratory for Stem Cell and Regenerative Medicine, Institute of Development and Regeneration, Guangzhou Institutes of Biomedicine and Health, Chinese Academy of Sciences, Guangzhou 510530, China
[4]Centre for Regenerative Medicine and Health, Hong Kong Institute of Science and Innovation, Chinese Academy of Sciences, 15 Science Park West Avenue, Hong Kong Science Park, Hong Kong SAR, China
[5]Shandong First Medical University & Shandong Academy of Medical Sciences, Jinan 250117, China
[6]Analytical and Instrumentation Core of Guangzhou Institutes of Biomedicine and Health, Chinese Academy of Sciences, Guangzhou 510530, China
[7]Madison West High School, Madsion, WI 53715, USA
[8]Lead contact
*Correspondence: zhu_yanling@gibh.ac.cn (Y.Z.), pan_guangjin@gibh.ac.cn (G.P.)
https://doi.org/10.1016/j.stemcr.2026.103048

SUMMARY

Natural Killer (NK) cells generally exhibit dysfunction in tumor microenvironment (TME), significantly limiting their efficacy in antitumor therapy. Tumor cells display enhanced glycolysis, leading to lactic acid (LA) secretion and accumulation in the TME. Using human-induced pluripotent stem cell (hiPSC)-derived NK cells (iNKs) as a model, we demonstrate that LA induces substantial iNK dysfunction, including reduced survival, impaired IFN-γ secretion, and diminished tumor-killing capacity due to severely compromised mitochondrial function. To overcome LA-induced dysfunction, we knocked in an expression cassette for lactate dehydrogenase B (LDHB) into hiPSCs (LDHB-hiPSCs), enabling conversion of cellular lactate to pyruvate. iNKs derived from LDHB-hiPSCs (LDHB-iNKs) largely resisted LA-induced dysfunction, exhibiting enhanced cytotoxicity and improved survival in high-LA tumor tissues. Importantly, LDHB-iNKs show superior suppression of solid tumor formation *in vivo*. Our study provides a novel strategy to enhance NK cell therapy against solid tumors by reshaping the metabolic pathways.

INTRODUCTION

NK cells are innate cytotoxic lymphocytes that spontaneously detect and kill infected or malignant cells based on integrated activating and inhibitory signals (Lanier 2008, Vivier et al., 2024). Unlike T cells, which require antigen presentation for cytotoxicity, NK cells eliminate tumor cells exhibiting "missing self" (loss of inhibitory ligands) or "induced self" (upregulation of activating ligands) (Chiossone et al., 2018; Myers and Miller 2021; Lanier 2024). Adoptive allogeneic NK cell transfer has been demonstrated to be safe, with low risks of graft-versus-host disease or cytokine release syndrome, enabling the "off-the-shelf" manufacturing for universal NK drug (Shimasaki et al., 2020; Myers and Miller 2021; Zhu et al., 2024). Particularly, human-induced pluripotent stem cell (hiPSC)-derived NK cells (iNKs) offer a homogeneous and scalable source for NK therapies (Fang et al., 2022; Xue et al., 2023). Many clinical studies have demonstrated the efficacy and potential of allogeneic NK cell therapies in treating hematological malignancies (Romee et al., 2016; Merino et al., 2023). Furthermore, genetically modified NK cells (e.g., chimeric antigen receptor [CAR]-NK cells) show positive efficacy against $CD19^+$ lymphoid malignancies (Liu et al., 2020; Marin et al., 2024). Recently, hiPSC-derived CAR-NK cells targeting $CD19^+$ B cells also demonstrated efficacy in autoimmune diseases (Wang et al., 2025). However, the efficacy of allogeneic NK therapies against solid tumors remains limited due to inefficient tumor homing and the immunosuppressive TME (Jia et al., 2023).

Many tumors exhibit accelerated glucose metabolism ("Warburg effect"), with upregulating glycolysis pathways. Enhanced glycolysis leads to co-export of lactate and protons, causing lactate acid (LA) accumulation in the TME (Wang et al., 2021). Elevated LA had been shown to correlate with tumor growth and metastasis (Wang et al., 2020). Accordingly, lactate dehydrogenase A (*LDHA*), which converts pyruvate to lactate, negatively correlates with survival in melanoma patients (Zheng et al., 2024). LA accumulation has been recognized to play an important role in immunosuppression. Melanomas with high *LDHA* expression show reduced infiltration and impaired function of T and NK cells (Brand et al., 2016). LA also limits dendritic

Published by Elsevier Inc. on behalf of International Society for Stem Cell Research.
This is an open access article under the CC BY license (http://creativecommons.org/licenses/by/4.0/).

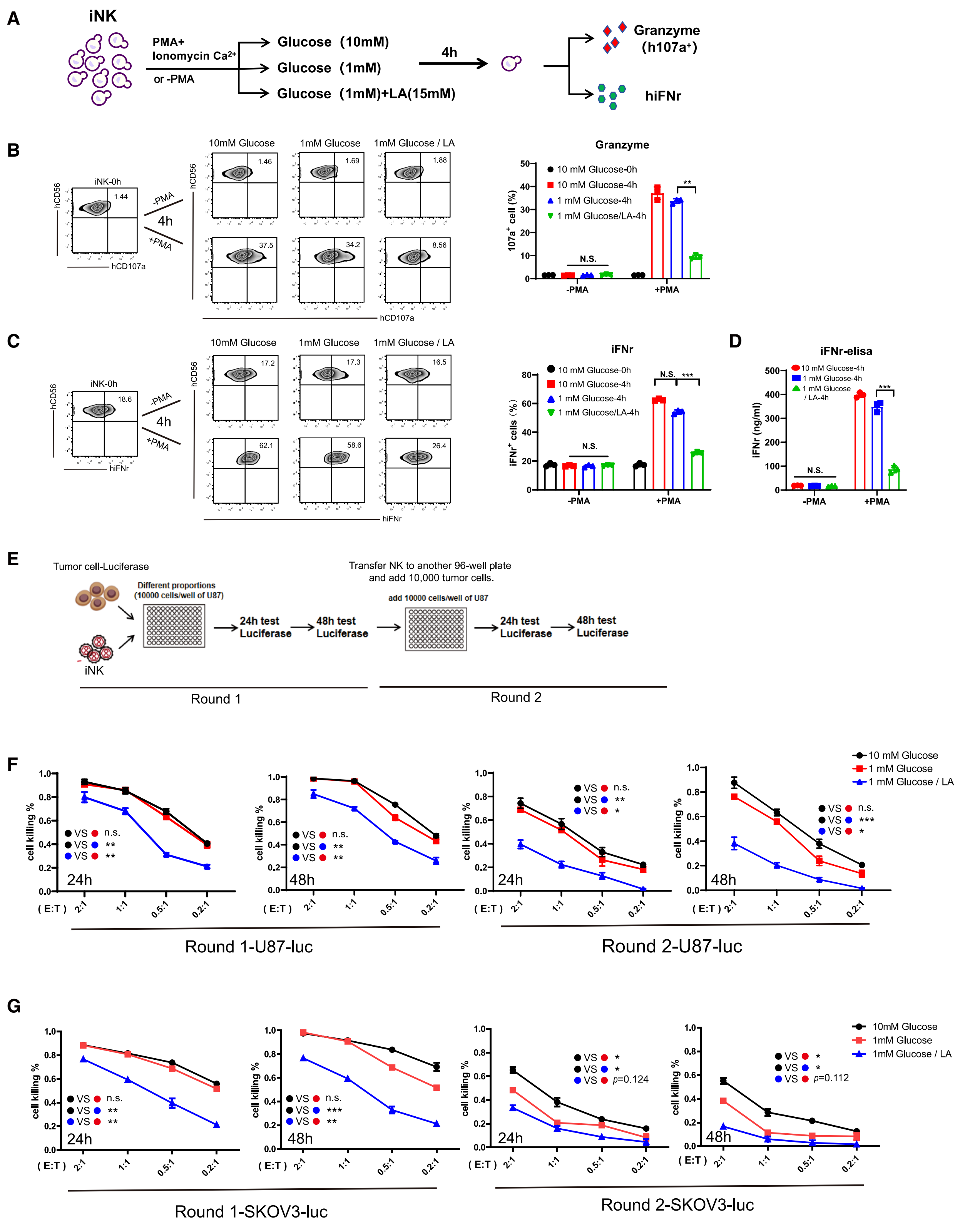

(legend on next page)

cell (DC) antigen presentation and migration (Plebanek et al., 2024). LA uptake by T cells causes intracellular acidification, impairing their metabolism and functions. How LA impacts NK cells remains poorly characterized.

Here, we analyze LA effects on iNKs, showing that LA substantially impairs mitochondria and induces dysfunctions in iNK cells. To counteract this, we generated iNKs stably expressing *LDHB* to convert the cellular lactate to pyruvate. LDHB-iNKs resisted LA-induced dysfunction and have enhanced survival in tumor tissue and superior tumor suppression capabilities.

RESULTS

LA induces dysfunction in iNK cells

iNKs hold great promise for off-the-shelf allogeneic cell therapy. Based on a defined monolayer approach that we previously developed for hematopoietic progenitor cells (iHSPCs) generation (Zhang et al., 2019), hiPSCs were firstly differentiated into multipotent hiHSPCs, followed by a two-step protocol to generate mature iNKs (Figure S1A). iHSPCs were co-cultured with OP9-DL1 to produce NK progenitors (iNKPs) which were then matured using modified K562 artificial antigen-presenting cells (APCs; Figure S1A) (Zhang et al., 2023). The generated iNKs expressed typical NK markers and receptors (Figure S1B). Upon PMA activation to trigger NK cytotoxicity, iNKs exhibited cytotoxic activities such as CD107a degranulation and IFN-γ secretion under high or low glucose conditions; however, 15 mM LA treatment significantly impaired these activities in iNKs (Figures 1A–1D). We then directly examined cytotoxic activity of iNKs against tumor cells in the presence of high concentration of LA (Figure 1E). Two different solid tumor cell lines, U87 and SKOV3 were selected for 2 rounds of repeated killing by iNKs at different effector-to-target (E:T) ratio in the presence or absence of 15 mM LA (Figure 1E). As shown in Figures 1F and 1G, iNKs showed slightly reduced tumor suppression under 1 mM glucose compared with 10 mM glucose but showed obvious impairment under 15 mM LA/1 mM glucose condition, particularly at lower E:T ratio and 2nd round of killing (Figures 1F and 1G). Together, these data indicate that LA severely impairs the cytotoxic activities of iNK cells.

LA impairs mitochondrial function in iNK cells

iNKs showed increased apoptosis under 15 mM LA/1 mM glucose during prolonged culture (Figure 2A). Given the importance of mitochondria in NK function and their susceptibility to LA, we assessed mitochondrial integrity in iNK treated by LA. Transmission electron microscopy (TEM) imaging revealed fewer and smaller mitochondria in iNKs under 15 mM LA/1 mM glucose vs. 10 mM glucose (Figure 2B). Immunostaining and western blot for the mitochondrial marker TOMM20 further confirmed the mitochondrial loss under LA treatment (Figures 2C and 2D). Based on JC-1 dye permeance assay, iNK cells under 15 mM LA/1 mM glucose condition displayed significantly greater mitochondrial depolarization (Figure 2E), indicating that LA induces dysfunction of mitochondria in iNK cells. Indeed, oxygen consumption rate (OCR) and extracellular acidification rate (ECAR) were reduced in iNK cells under 15 mM LA/1 mM glucose condition (Figure 2F). Consistently, ATP production as well as NAD^+/NADH ratio was also reduced in iNK cells treated by 15 mM LA (Figure 2G). Together, these data demonstrate that higher concentration of LA impaired mitochondria functions in iNK cells.

Generation of *LDHB*-expressing hiPSCs via gene targeting

LDHA and DHB are two major enzymes involved in lactate metabolism, mediating bidirectional conversion between pyruvate and lactate. LDHA preferentially converts pyruvate to lactate, while LDHB favors lactate-to-pyruvate conversion. Based on quantitative reverse-transcription PCR (RT-qPCR), both undifferentiated hiPSCs and iHSPCs showed higher *LDHB* expression than *LDHA* while iNK had lower *LDHB* expression than *LDHA* (Figure 3A). To confer LA resistance, we knocked in an *LDHB* expression cassette into the ROSA26 locus of hiPSCs

Figure 1. Lactate impairs the effector functions of iNK cells

(A) Schematic of the experimental design for cytokine stimulation. iNKs were stimulated with PMA and ionomycin for 4 h under the indicated glucose/lactate conditions, followed by evaluation of IFN-γ and granzyme B production.

(B and C) Flow cytometry analysis of IFN-γ and granzyme B expression in iNK cells under different treatment conditions. *N = 3* per group for from three independent differentiation batches.

(D) Secreted IFN-γ levels in iNK culture supernatants under the indicated conditions, as measured by ELISA. *N = 3* per group for from three independent differentiation batches.

(E) Schematic of the iNK cytotoxicity assay.

(F and G) Cytotoxic activity of iNKs against the indicated tumor cell lines under different metabolic conditions. *N = 3* per group for from three independent differentiation batches. Statistical analysis: two-way ANOVA followed by Tukey's test; $*p < 0.05$, $**p < 0.01$, $***p < 0.001$.

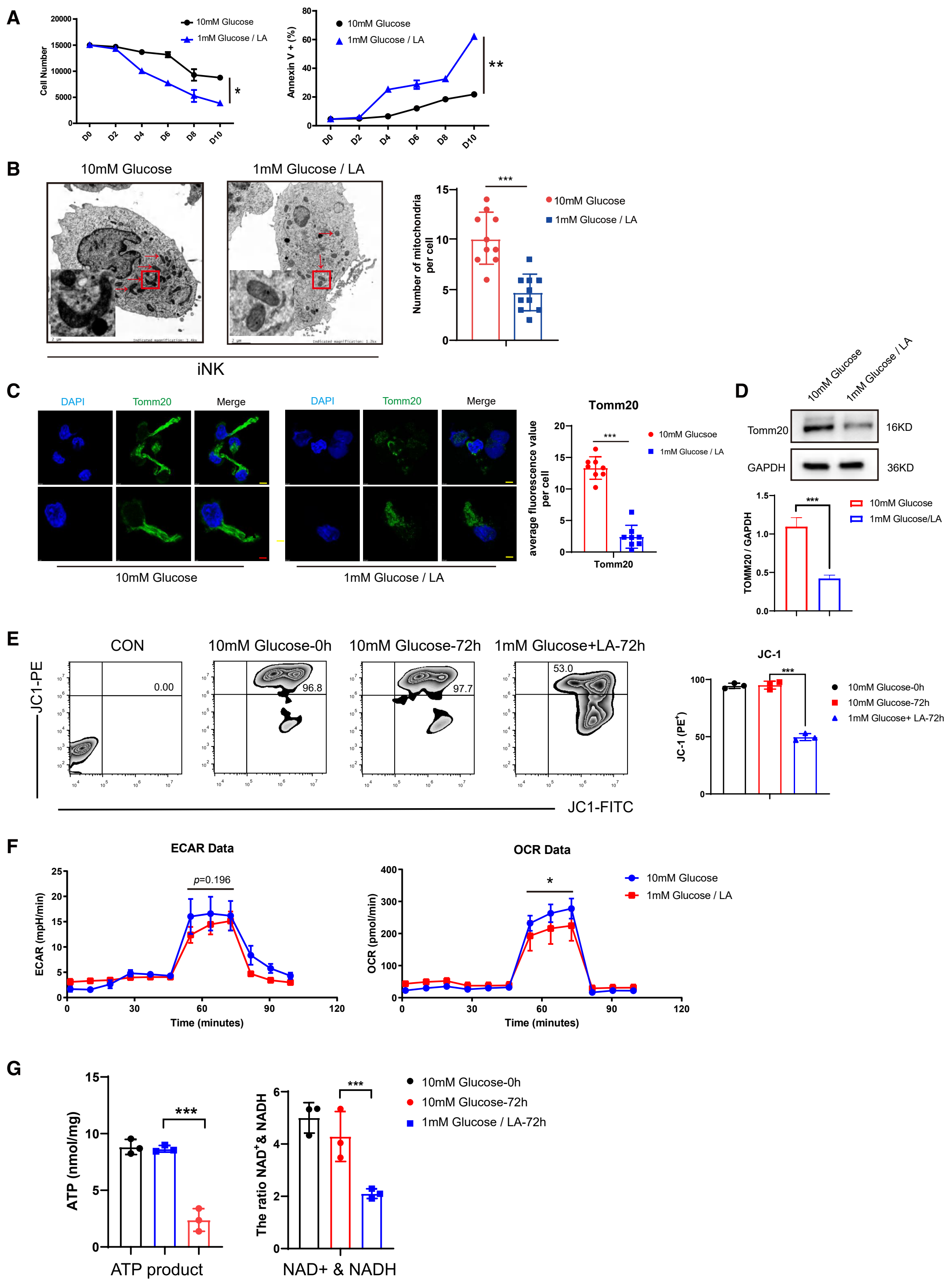

(legend on next page)

using CRISPR-Cas9-aided homologous recombination (Figure 3B). LDHB-hiPSCs exhibited normal karyotype (Figure S2A) and elevated *LDHB* expression while maintaining expression of pluripotency genes (*OCT4*, *SOX2*, and *NANOG*) without aberrant lineage commitment (Figures 3C–3F) and enhanced proliferation (Figure 3G). To test the role of LDHB, we then treated hiPSCs with 15 mM LA or together with 2-Deoxy-D-glucose (2DG), a glucose analog to block glucose uptake (Figures 3H and 3I). LA treatment induced massive cell death in wild-type (WT) hiPSCs while had little effect on hiPSCs_LDHB (Figures 3H and 3I). Moreover, hiPSCs_LDHB also showed a higher survival rate than WT hiPSCs under 15 mM LA/2DG condition (Figures 3H–3J; Figure S2B). In all, we generate hiPSCs with stably expressing *LDHB* that protects the LA-induced cell death.

LDHB-hiPSCs exhibit enhanced iHSPC generation efficiency

We then examined the efficiency of iHSPC generation by hiPSCs-LDHB based on our previously published stepwise, mono-layer-based differentiation protocol (Zhang et al., 2019; Zhu et al., 2020) (Figure 4A). Based on this protocol, iHSPCs were generated from the attached endothelia cells and became floated in the medium. We calculated the produced iHSPCs floating in the medium every two days in 1 well of 6-well plate differentiation culture (Figure 4B). hiPSCs_LDHB showed more iHSPC yield compared with WT hiPSCs, particularly at the late stages of differentiation (Figure 4B). Both hiPSCs_LDHB or WT hiPSCs-derived iHSPCs expressed typical iHPSC markers, such as CD43 and CD45 (Figure 4C). As expected, iHSPCs derived from hiPSCs_LDHB maintained higher expression of *LDHB* and showed much more resistant to LA-induced cell death (Figures 4D and 4E; Figures S3A and S3B). Based on colony forming unit (CFU) assay, hiPSCs_LDHB-derived iHSPCs showed similar formation of different blood lineage CFUs compared with WT cells (Figure 4F). Together, these data indicate that LDHB-hiPSCs hold the potential to produce more iHSPCs that resist the LA-induced cell death.

LDHB-iNKs resist LA-induced mitochondrial dysfunction and death

We then generated iNK cells from hiPSCs_LDHB-derived iHSPCs. LDHB-iNK cells showed typical NK morphology and maintained higher expression of *LDHB* (Figures 5A and 5B). The NK-specific inhibitory and activating receptors were well expressed in LDHB-iNK cells with similar pattern to WT iNK cells (Figure 5C), indicating *LDHB* expression did not impact iNK generation. Upon prolonged *in vitro* culture, LDHB-iNK maintained higher cell numbers compared with WT iNK under condition of 15 mM LA (Figures 5D and 5E). Western blot on TOMM2, the mitochondrial outer membrane protein, showed that LDHB-iNK resisted the mitochondrial loss induced by 15 mM LA (Figure 5F). LA induced mitochondrial depolarization as well as reduction of ATP and NAD^+ were also well restored in LDHB-iNK cells compared with WT iNK cells (Figures 5G–5I). Together, these data demonstrate that LDHB expression rescues LA-induced mitochondrial dysfunction in iNK cells.

LDHB-iNKs display enhanced cytotoxicity

We then examined cytotoxicity of LDHB-iNK cells under condition with or without LA. Compared with WT iNK cells, LDHB-iNK cells restored the LA-impaired granzyme (CD107a) delivery and IFN-γ secretion upon PMA activation (Figures 6A–6C; Figure S4A). We then directly examined cytotoxic activity of LDHB-iNK against tumor cells under different conditions with or without LA at different concentrations (Figure 6D).

As expected, under 15 mM LA condition, LDHB-iNK exhibited much higher cytotoxicity to kill different tumor

Figure 2. Lactate impairs mitochondrial function in iNK cells

(A) Proliferation and apoptosis rates of iNKs cultured for 10 days in normal (10 mM glucose) or TME-simulating (1 mM glucose +15 mM lactate) conditions, assessed every two days. *N = 3* per group for from three independent differentiation batches. Statistical analysis: two-way ANOVA followed by Tukey's test; $*p < 0.05$, $**p < 0.01$.

(B) Representative electron microscopy images of mitochondria in iNKs treated for 4 days with 15 mM glucose or 15 mM lactate/1 mM glucose. Scale bars, 1 μm. *N = 10*, the quantity is derived from the number of mitochondria in each cell.

(C) Confocal microscopy images showing mitochondrial morphology (TOMM20, green; nucleus, blue) in iNKs treated as in (B). Scale bars, 10 μm. Fluorescence intensity of TOMM20 was quantified using ImageJ. *N = 8*, the fluorescence intensity is derived from 8 different fields of view in each group. Scale bars (yellow), 5 μm; (red), 2 μm.

(D) Western blot analysis of TOMM20 expression in iNKs under the indicated treatments. *N = 3*, data are from three independent differentiation batches.

(E) Flow cytometry analysis of mitochondrial membrane potential (JC-1 staining, PE^+ intensity) in iNKs after treatment. *N = 3*, data are from three independent differentiation batches.

(F)Seahorse assay profiles of extracellular acidification rate (ECAR) and oxygen consumption rate (OCR) in iNKs treated for 3 days with 15 mM glucose or 15 mM lactate/1 mM glucose. *N = 3* per group for from three independent differentiation batches.

(G) Intracellular ATP levels and NAD/NADH ratios in treated iNKs. For (E)–(G), iNKs were treated for 3 days. *N = 3*, data are from three independent differentiation batches. Data are mean ± SD; $*p < 0.05$, $**p < 0.01$, $***p < 0.001$.

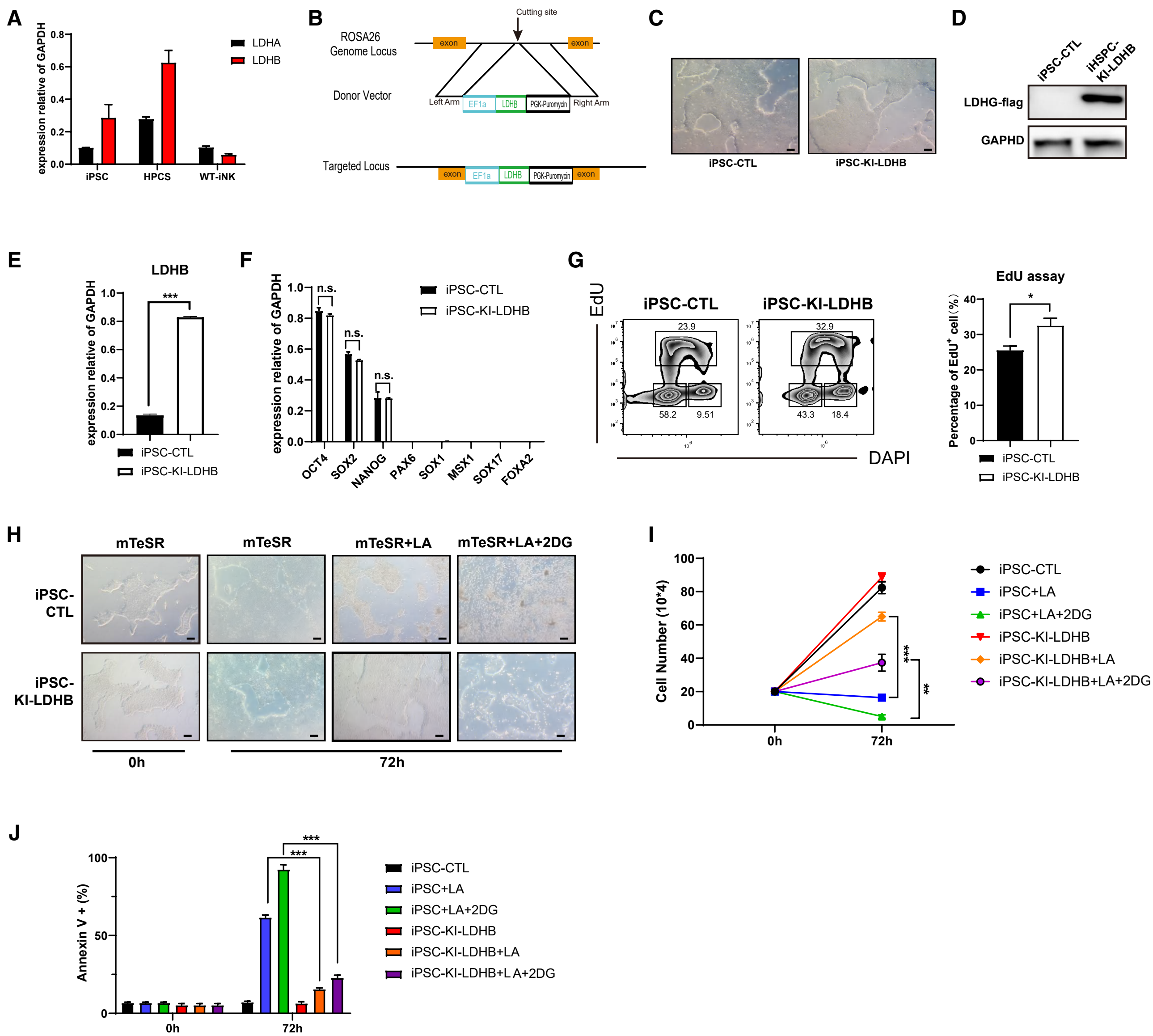

Figure 3. Generation and characterization of *LDHB*-expressing hiPSCs

(A) RT-qPCR analysis of *LDHA* and *LDHB* expression during differentiation from iPSCs to HPCs to iNKs.

(B) Schematic of the targeting strategy for LDHB knock-in at the ROSA26 locus.

(C) Morphology of wild-type (WT) and LDHB-knock-in (LDHB-hiPSCs) iPSC colonies. Scale bars, 200 μm.

(D and E)(D) Western blot and (E) RT-qPCR analysis of LDHB expression in WT and LDHB-hiPSCs. *N = 3*, data are from three independent differentiation batches.

(F) RT-qPCR analysis of pluripotency markers (*OCT4*, *SOX2*, and *NANOG*) and germ layer markers (*PAX6*, *SOX1*, *MSX1*, *SOX17*, and *FOXA2*) in WT and LDHB-hiPSCs. *N = 3* per group for from three independent differentiation batches.

(G) Cell-cycle analysis by EdU incorporation; quantification of S-phase (EdU^+) cells is shown. *N = 3*, data are from three independent differentiation batches.

(H) Morphology of WT and LDHB-hiPSCs after 72-h culture in mTeSR medium alone, or supplemented with lactate, or lactate + the glycolytic inhibitor 2-Deoxy-D-glucose (2-DG). Scale bars, 200 μm.

(I and J) (I) Cell count and (J) apoptosis analysis for cells in (H). *N = 3*, data are from three independent differentiation batches. Data are mean ± SD; $*p < 0.05$, $**p < 0.01$, $***p < 0.001$.

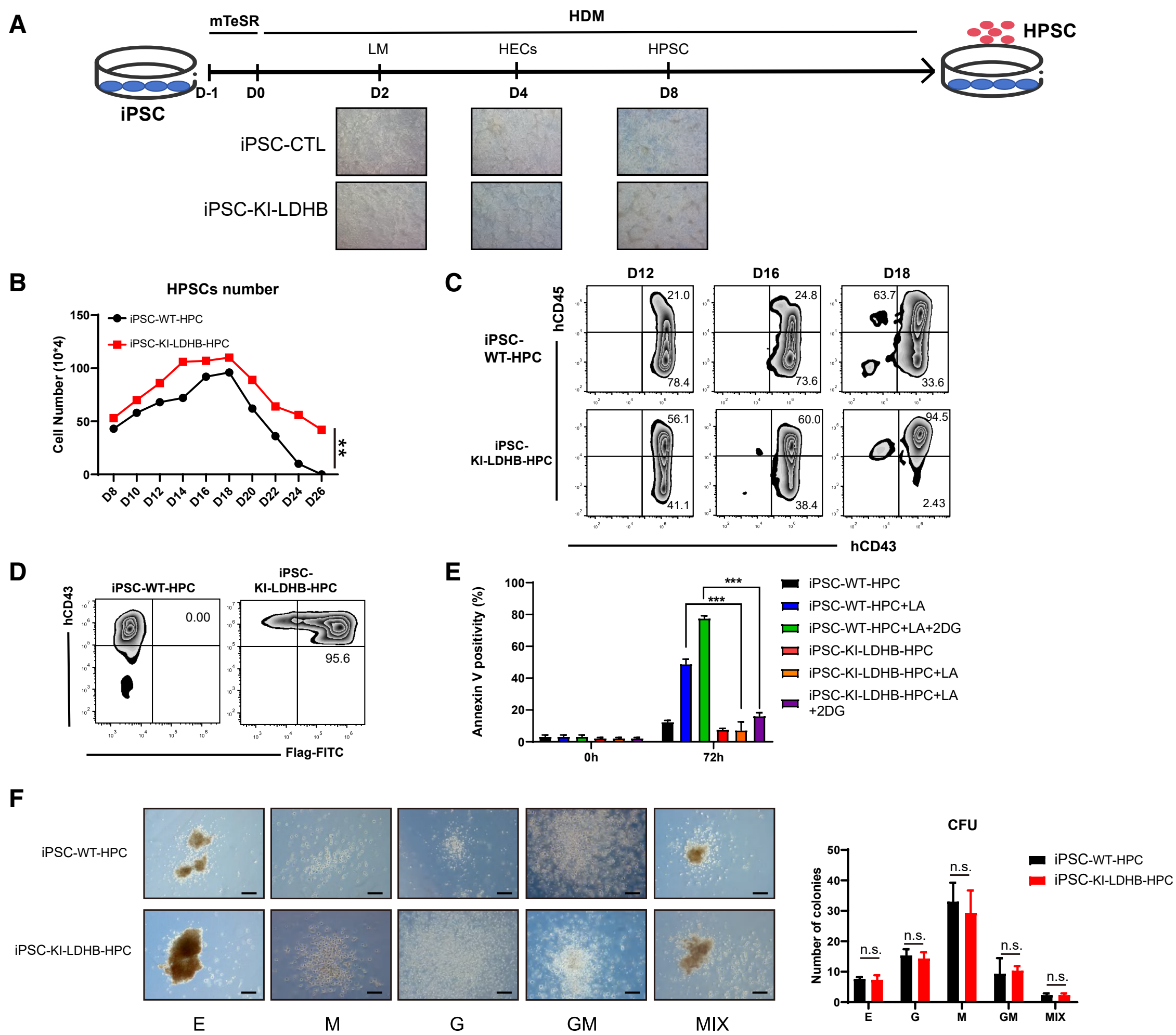

Figure 4. LDHB-hiPSCs demonstrate enhanced efficiency in generating hematopoietic progenitor cells

(A) Schematic of the differentiation protocol from iPSCs to hematopoietic progenitor cells (iHSPCs).

(B) Quantification of iHSPCs produced during hematopoietic differentiation from day 8 to day 28. $N = 3$ per group for from three independent differentiation batches. Statistical analysis: two-way ANOVA followed by Tukey's test; $^{**}p < 0.01$.

(C) Expression of hematopoietic markers CD43 and CD45 on iHSPCs derived from WT or LDHB-hiPSCs.

(D) Flow cytometry analysis of CD43 and LDHB (FLAG tag) expression in day 12 iHSPCs.

(E) Apoptosis in WT and LDHB_iHSPCs after 72-h culture in differentiation medium alone, or with lactate, or lactate + 2-DG. $N = 3$ per group for from three independent differentiation batches.

(F) Representative images of colony-forming unit (CFU) assays from sorted iHSPCs (day 10). Scale bars, 100 μm. Colonies were classified as erythroid (E), granulocyte (G), macrophage (M), granulocyte-macrophage (GM), or mixed (Mix). The bar graph shows quantitative analysis of CFU types. $N = 3$ per group for from three independent differentiation batches. Data are mean ± SD; $^{*}p < 0.05$, $^{**}p < 0.01$, $^{***}p < 0.001$. Scale bars, 50 μm.

cells such as U87 and SKOV3 compared with WT iNKs (Figure 6D). Notably, in the absence of LA, LDHB-iNK also showed enhanced tumor cell suppression under 10 mM glucose condition, particularly at lower E:T ratio and 2nd round killing (Figure 6D), indicating LDHB-iNK cells might have more resistance to exhaustion. We also examined the expression of exhaustion-related genes in iNK cells during multiple rounds of killing. The results showed that *LDHB* overexpression reduced the expression of the exhaustion marker TIM-3 on the surface of iNK cells after repeated killing, compared with WT iNK cells (Figure S4B). We also examined the cytotoxicity of LDHB-iNK cells under low glucose condition (1 mM) in the presence or absence of low concentration of LA (5 mM). Interestingly, while low concentration of LA (5 mM) slightly suppressed WT iNK cells in tumor killing, it had a promoting effect on LDHB-iNK cells, particularly at 2nd round of tumor killing (Figure 6E).

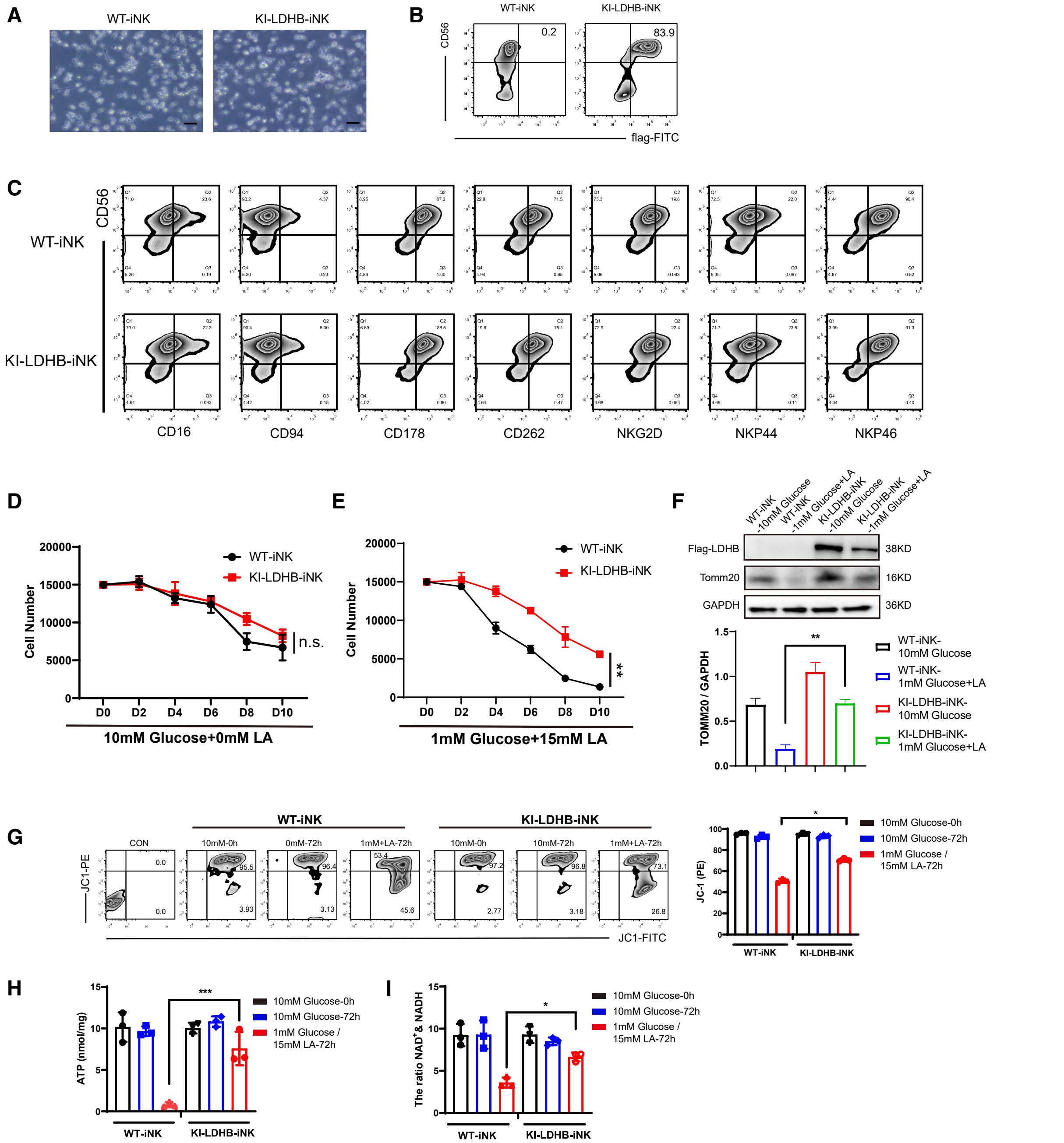

Figure 5. LDHB-iNKs are resistant to lactate-induced mitochondrial dysfunction and cell death

(A) Morphology of WT iNKs and LDHB-iNKs. Scale bars, 20 μm.

(B) Flow cytometry analysis of CD56 and LDHB (FLAG tag) expression.

(C) Expression profiles of key inhibitory and activating NK cell receptors.

(D and E) Quantification of WT and LDHB-iNK cell numbers during 10-day culture in normal (10 mM glucose) or TME-simulating (1 mM glucose +15 mM lactate) conditions. *N = 3* per group for from three independent differentiation batches. Statistical analysis: two-way ANOVA followed by Tukey's test; $*p < 0.05$, $**p < 0.01$, $***p < 0.001$.

(F) Western blot analysis of TOMM20 expression in WT and LDHB-iNKs with or without lactate exposure. *N = 3* per group for from three independent differentiation batches.

(legend continued on next page)

LA is usually transported by monocarboxylate transporters, such as MCT1 and MCT4. Indeed, inhibition of MCT-1 by the MCT1-specific inhibitor AZD3965 reduced the intracellular lactate levels (Figure S4C). LDHB overexpression did not significantly alter the mRNA levels of MCT1 and MCT4 on iNK cells (Figure S4D) but showed further suppression on intracellular lactate in AZD3965-treated iNKs (Figure S4C). The intracellular pyruvate-to-lactate ratio increased in LDHB-expressing iNK cells compared to controls under PMA stimulation (Figure 6F), indicating LDHB promotes iNK function mainly through metabolic reprogramming rather than suppressing LA uptake. Indeed, ATP production impaired by LA was rescued in LDHB-expressing iNK cells (Figure 6G). In addition, iNK cells treated by a sodium-hydrogen exchanger inhibitor EIPA to induce accumulation of intracellular H^+ and acidosis showed less impact on tumor killing (Figures S4E and S4F). In contrast, iNK cells treated by sodium lactate showed significant reduction of tumor killings, which were rescued by LDHB (Figure S4G). Together, we demonstrate that LDHB promotes the function of iNK cells through a metabolic reprogramming on lactate.

LDHB-iNKs exhibit enhanced survival in tumors and superior antitumor activity

We then examined the survival of LDHB-iNK cells in solid tumor tissue that have high LA concentration. We firstly performed subcutaneous injection of U87 tumor cells in mice to form solid tumor tissues and then injected LDHB-iNK or WT iNK cells into the tumor tissue (Figure 7A). The survival of NK cells were analyzed 72 h later. As expected, tumor tissue showed much more elevated LA compared with other normal organ tissues (Figure 7B). There was no significant difference in tumor weight between tumors injected with WT or LDHB-iNK (Figure 7C). However, LDHB-iNK cells showed much more survival in tumors compared with WT iNK cells (Figure 7D). We then examined *in vivo* suppression of tumor formation by WT or LDHB-iNK cells (Figure 7E). U87 tumor cells were subcutaneous injected and the WT iNK or LDHB-iNK cells were injected 3 days later for total 3 doses with 4 days interval (Figure 7E). Tumor growth was monitored by fluorescence as indicated (Figure 7E; Figure S5A). There was no significant difference in the weight among the mice in each group during the experiment (Figure S5B). Tumor volumes were calculated at the end of the experiment. As shown in Figures 7F and 7G, WT iNK injection showed significant suppression on tumor formation and growth while LDHB-iNK displayed much more enhanced suppression effects (Figures 7F–7H). These data demonstrate that LDHB-iNK cells have enhanced survival in tissue and better anti-tumor potency.

DISCUSSION

The efficacy of adoptive NK cell therapy against solid tumors is hindered by the immunosuppressive TME (Wu et al., 2020). Accumulation of LA in TME has been recognized as an important factor promoting tumor growth and progression through suppressing T cells and NK cells (Brand et al., 2016; Jiang et al., 2024). Here, in this study, we reveal that expression of *LDHB* in stem cell-derived NK cells not only overcomes LA-induced dysfunctions but also promotes their tumor-suppression functions. LDHB_NK cells display enhanced cytotoxicity against different tumor cells, particularly at low E:T ratios or in the presence of low concentration of LA. Moreover, LDHB-iNK cells display better survival in tumor tissue and enhanced suppression on tumor formation based on mice model.

It is clear that tumor cells secreted LA that plays an essential role to regulate the invading immune cells. LA has been shown to induce lysine lactylation (Kla) in NK cells and mitochondria fragmentation, leading to a reduced cytotoxicity (Jin et al., 2025). How exactly LA impaired mitochondria structure and function remains unclear. Continuous exposure to LA also impaired functions of other immune cells, such as T cells, DC cells etc (Plebanek et al., 2024). Lactate production is a critical and highly regulated step in glycolysis, which controlled by LDH. LDH have five isoenzymes (LDH1–5) comprised by LDHA and LDHB, mediating the bidirectional conversion of pyruvate and lactate. LDHA has a higher affinity for pyruvate and preferentially converts pyruvate to lactate while LDHB has more affinity for lactate and mainly convert lactate to pyruvate (Urbanska and Orzechowski 2019). LDH divergence provides a regulatory mechanism to specifically control the metabolic status in a particular tissue or cell type. During iNK differentiation, both hiPSCs and iHPCs have a relatively higher LDHB expression while iNK cells have a lower LDHB expression (Figure 3A). Over-expression of LDHB not only benefits the *in vitro* culture of hiPSCs (Figure 3) and also promotes iHPCs generation (Figure 4). Interestingly, LDHB-iNK cells display an enhanced cytotoxicity in the presence of low concentration of LA (Figure 5). The LA

(G) Flow cytometry analysis of mitochondrial membrane potential (JC-1) with or without lactate exposure; quantification of JC-1 (PE^+) intensity is shown. *N = 3*, data are from three independent differentiation batches.
(H and I) Intracellular ATP production and NAD/NADH ratios in WT and LDHB-iNKs with or without lactate exposure. *N = 3*, data are from three independent differentiation batches. Data are mean ± SD; *$p < 0.05$, **$p < 0.01$, ***$p < 0.001$.

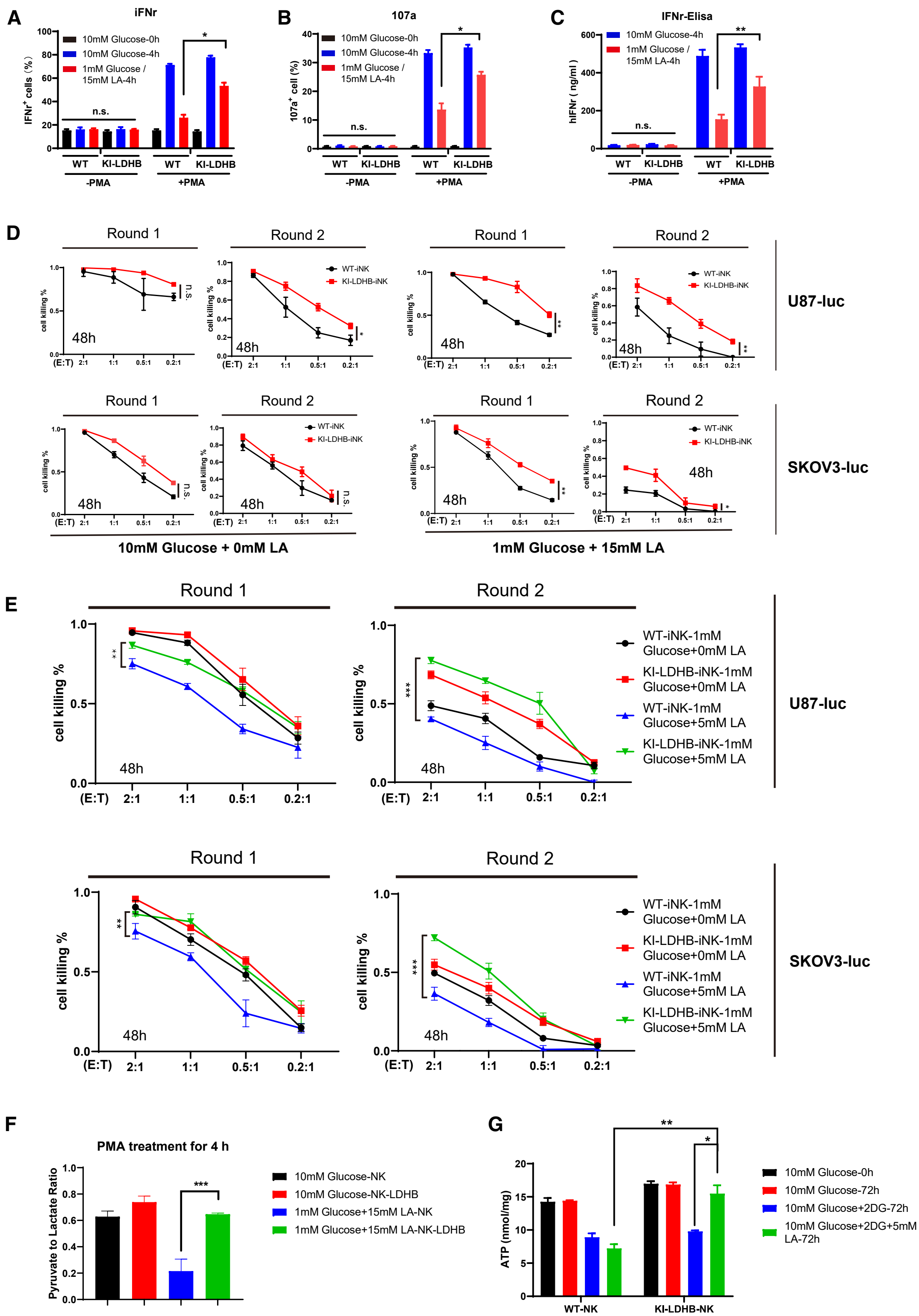

(legend on next page)

taken in LDHB-iNK cells could be converted to pyruvate to be further used for citric-acid cycle for providing additional ATP (Figure 5). Thus, our studies provide a novel strategy to improve NK cell therapy against solid tumors through reshaping the LDH isoenzyme spectrum.

RESOURCE AVAILABILITY

Lead contact

Requests for further information and resources should be directed to and will be fulfilled by the lead contact, Guangjin, Pan (pan_guangjin@gibh.ac.cn).

Materials availability

No new materials were generated in this study.

Data and code availability

No new custom code was generated in this study; however, the scripts used for data analysis are available from the corresponding author upon reasonable request.

ACKNOWLEDGMENTS

This work was supported by the Strategic Priority Research Program of Chinese Academy of Sciences (grant no. XDB0940301); Major Research Project of Guangzhou Institutes of Biomedicine and Health, Chinese Academy of Sciences(GIBHMRP25-01); the National Key Research and Development Program of China (2022YFA1105001); the Research Funds from Health@InnoHK Program launched by Innovation Technology Commission of the Hong Kong SAR, P. R. China; the National Natural Science Foundation of China (32171451 and 32270624); Guangdong Provincial Key Laboratory of Stem Cell and Regenerative Medicine (2020B1212060052, 2023B1212060050, and 2023B1212120009); Guangzhou Science and Technology Program General project (202206010041 and 2024A04J3801); the Youth Innovation Promotion Association of the Chinese Academy of Sciences (2023373); Major Project of Guangzhou National Laboratory (GZNL2024A03012); and Guangdong Provincial Key Laboratory of Stem Cell and Regenerative Medicine, Guangdong-Hong Kong Joint Laboratory for Stem Cell and Regenerative Medicine.

AUTHOR CONTRIBUTIONS

G.P. and Y.Z. initiated and designed the project. G.P., Y.Z., and Z.Z. wrote the manuscript. Z.Z. performed most experiments and analyzed the data. Y.M., J.T., and J.L. performed the iNK differentiation experiments and analyzed result data. J.N. and Z.Z. constructed the plasmid and detected. Y.Q. constructed the knocked-in cell line. Z.Z. and Z.C. completed the animal experiments. J.G, T.Z. J.W., J.P, and J.C. gave experiment suggestions or provided experiment materials for this research. All authors read and approved the final manuscript.

DECLARATION OF INTERESTS

All authors declare no conflict of interest.

STAR★METHODS

Detailed methods are provided in the online version of this paper and include the following:

- KEY RESOURCES TABLE
- EXPERIMENTAL MODEL AND STUDY PARTICIPANT DETAILS
  - Cell lines and cell culture
  - Mice
  - Hematopoietic differentiation with hESCs
  - Natural killer cells differentiation
  - Gene targeting
- METHOD DETAILS
  - CFU assay
  - RT-qPCR
  - Flow cytometry
  - Cell-cycle and apoptosis analysis
  - Immunofluorescence
  - Western Blot
  - Measurement of intracellular pyruvate and lactate levels
  - Intracellular pH (pHi) measurement
  - *In vitro* cytotoxicity assay
  - Animal experiments
  - Statistical analysis

Figure 6. LDHB-iNKs exhibit enhanced cytotoxic function
(A and B) Flow cytometry analysis of IFN-γ and granzyme B secretion in WT and LDHB-iNKs, with or without lactate exposure, following 4-h PMA/ionomycin stimulation. $N = 3$, data are from three independent differentiation batches.
(C) ELISA quantification of IFN-γ secretion in culture supernatants. $N = 3$, data are from three independent differentiation batches.
(D) Cytotoxicity of WT and LDHB-iNKs against luciferase-labeled U87 (glioblastoma) or SKOV3 (ovarian cancer) cell lines at indicated effector-to-target (E:T) ratios, measured every 48 h in normal (10 mM glucose) or TME-simulating (1 mM glucose +15 mM lactate) conditions. $N = 3$ per group for from three independent differentiation batches. Statistical analysis: two-way ANOVA followed by Tukey's test; $*p < 0.05$, $**p < 0.01$, $***p < 0.001$.
(E) Cytotoxicity under low glucose (1 mM) with or without low lactate (5 mM). $N = 3$ per group for from three independent differentiation batches. Statistical analysis: two-way ANOVA followed by Tukey's test.; $*p < 0.05$, $**p < 0.01$, $***p < 0.001$.
(F) The pyruvate-to-lactate ratio in NK cells under different metabolic conditions. $N = 3$, data are from three independent differentiation batches. Data are mean ± SD.
(G) Intracellular ATP production in WT and LDHB-iNKs with or without low lactate (5 mM) exposure. $N = 3$, data are from three independent differentiation batches. Statistical analysis: two-way ANOVA followed by Tukey's test.; $*p < 0.05$, $**p < 0.01$, $***p < 0.001$.

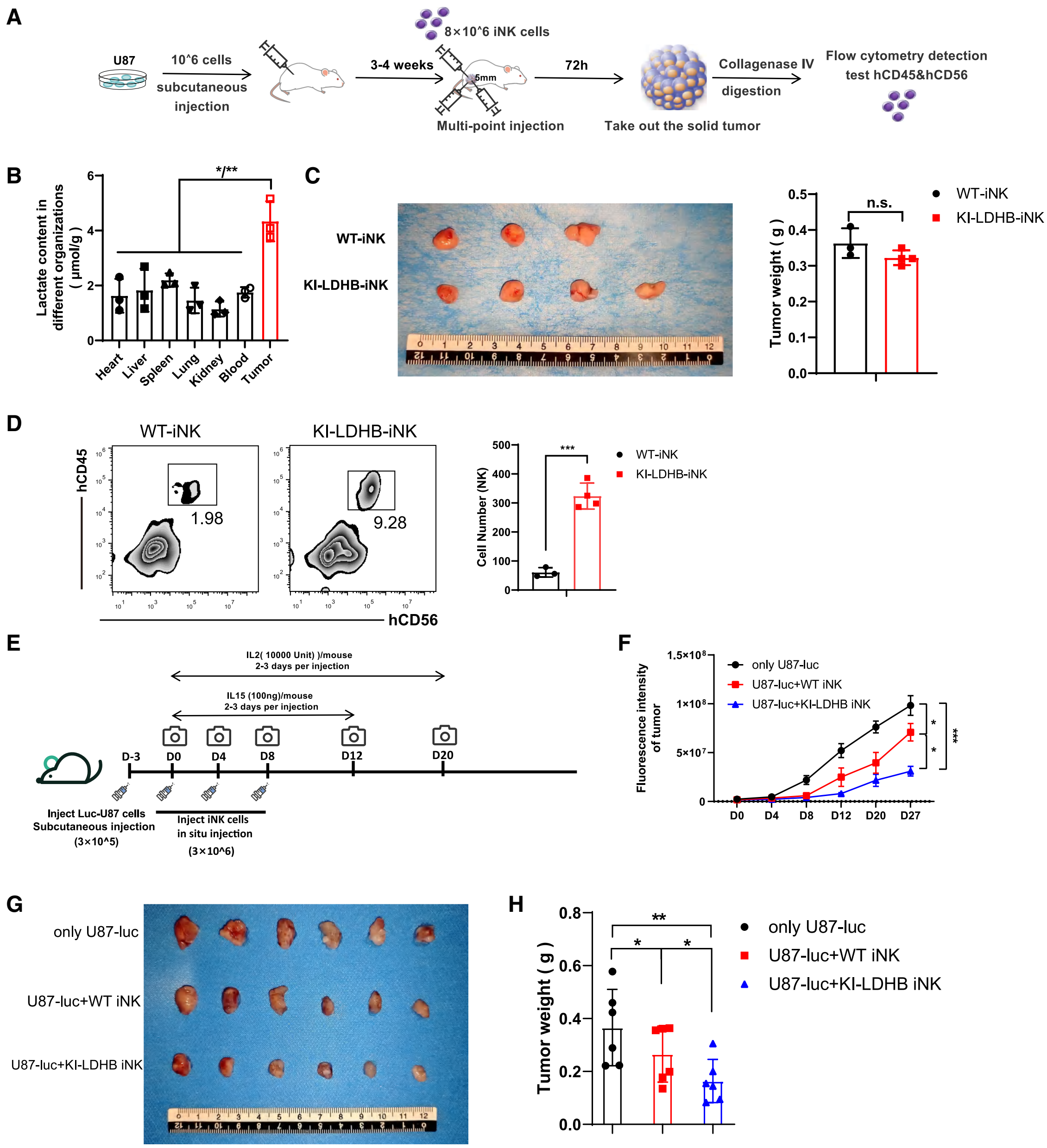

Figure 7. LDHB-iNKs show improved tumor retention and superior antitumor efficacy *in vivo*

(A) Schematic of the experiment to quantify iNK cell survival in U87 tumors 72 h post-injection.

(B) Lactate concentrations measured in mouse organs and tumors 72 h after injection of WT iNKs. *N = 3*, with each set of data sourced from different mice.

(C) Weight of excised tumors from the experiment in (A) and (B).

(D) Flow cytometry quantification of human iNKs ($hCD45^+$ and $hCD56^+$) in enzymatically dissociated (collagenase IV) tumors. *N* = 3 or 4, with each set of data sourced from different mice.

(E) Schematic of the *in vivo* tumor treatment model. U87-luc cells were implanted subcutaneously, followed by three doses of iNKs every 4 days.

(F) *In vivo* bioluminescence imaging to monitor tumor burden in mice treated with WT or LDHB-iNKs over time. *N = 3*, with each set of data sourced from different mice. Statistical analysis: two-way ANOVA followed by Tukey's test; $*p < 0.05$, $**p < 0.01$, $***p < 0.001$.

(G) Representative photographs of excised tumors at endpoint (day 27).

(H) Weight of tumors from (G). *N = 6*, each set of data is sourced from different mice, with each mouse contributing 2 tumor tissues. Data are mean ± SD; $*p < 0.05$, $**p < 0.01$, $***p < 0.001$.

SUPPLEMENTAL INFORMATION

Supplemental information can be found online at https://doi.org/10.1016/j.stemcr.2026.103048.

Received: February 2, 2026
Revised: July 14, 2026
Accepted: July 14, 2026
Published: August 13, 2026

REFERENCES

Brand, A., Singer, K., Koehl, G.E., Kolitzus, M., Schoenhammer, G., Thiel, A., Matos, C., Bruss, C., Klobuch, S., Peter, K., et al. (2016). LDHA-Associated Lactic Acid Production Blunts Tumor Immunosurveillance by T and NK Cells. Cell Metab. *24*, 657–671. https://doi.org/10.1016/j.cmet.2016.08.011.

Chiossone, L., Dumas, P.Y., Vienne, M., and Vivier, E. (2018). Natural killer cells and other innate lymphoid cells in cancer. Nat. Rev. Immunol. *18*, 671–688. https://doi.org/10.1038/s41577-018-0061-z.

Fang, F., Xie, S., Chen, M., Li, Y., Yue, J., Ma, J., Shu, X., He, Y., Xiao, W., and Tian, Z. (2022). Advances in NK cell production. Cell. Mol. Immunol. *19*, 460–481. https://doi.org/10.1038/s41423-021-00808-3.

Jia, H., Yang, H., Xiong, H., and Luo, K.Q. (2023). NK cell exhaustion in the tumor microenvironment. Front. Immunol. *14*, 1303605. https://doi.org/10.3389/fimmu.2023.1303605.

Jiang, M., Wang, Y., Zhao, X., and Yu, J. (2024). From metabolic byproduct to immune modulator: the role of lactate in tumor immune escape. Front. Immunol. *15*, 1492050. https://doi.org/10.3389/fimmu.2024.1492050.

Jin, J., Yan, P., Wang, D., Bai, L., Liang, H., Zhu, X., Zhu, H., Ding, C., Wei, H., and Wang, Y. (2025). Targeting lactylation reinforces NK cell cytotoxicity within the tumor microenvironment. Nat. Immunol. *26*, 1099–1112. https://doi.org/10.1038/s41590-025-02178-8.

Lanier, L.L. (2008). Up on the tightrope: natural killer cell activation and inhibition. Nat. Immunol. *9*, 495–502. https://doi.org/10.1038/ni1581.

Lanier, L.L. (2024). Five decades of natural killer cell discovery. J. Exp. Med. *221*, e20231222. https://doi.org/10.1084/jem.20231222.

Liu, E., Marin, D., Banerjee, P., Macapinlac, H.A., Thompson, P., Basar, R., Nassif Kerbauy, L., Overman, B., Thall, P., Kaplan, M., et al. (2020). Use of CAR-Transduced Natural Killer Cells in CD19-Positive Lymphoid Tumors. N. Engl. J. Med. *382*, 545–553. https://doi.org/10.1056/NEJMoa1910607.

Marin, D., Li, Y., Basar, R., Rafei, H., Daher, M., Dou, J., Mohanty, V., Dede, M., Nieto, Y., Uprety, N., et al. (2024). Safety, efficacy and determinants of response of allogeneic CD19-specific CAR-NK cells in CD19(+) B cell tumors: a phase 1/2 trial. Nat. Med. *30*, 772–784. https://doi.org/10.1038/s41591-023-02785-8.

Merino, A., Maakaron, J., and Bachanova, V. (2023). Advances in NK cell therapy for hematologic malignancies: NK source, persistence and tumor targeting. Blood Rev. *60*, 101073. https://doi.org/10.1016/j.blre.2023.101073.

Myers, J.A., and Miller, J.S. (2021). Exploring the NK cell platform for cancer immunotherapy. Nat. Rev. Clin. Oncol. *18*, 85–100. https://doi.org/10.1038/s41571-020-0426-7.

Plebanek, M.P., Xue, Y., Nguyen, Y.V., DeVito, N.C., Wang, X., Holtzhausen, A., Beasley, G.M., Theivanthiran, B., and Hanks, B.A. (2024). A lactate-SREBP2 signaling axis drives tolerogenic dendritic cell maturation and promotes cancer progression. Sci. Immunol. *9*, eadi4191. https://doi.org/10.1126/sciimmunol.adi4191.

Romee, R., Rosario, M., Berrien-Elliott, M.M., Wagner, J.A., Jewell, B.A., Schappe, T., Leong, J.W., Abdel-Latif, S., Schneider, S.E., Willey, S., et al. (2016). Cytokine-induced memory-like natural killer cells exhibit enhanced responses against myeloid leukemia. Sci. Transl. Med. *8*, 357ra123. https://doi.org/10.1126/scitranslmed.aaf2341.

Shan, Y., Zhang, Y., Zhao, Y., Wang, T., Zhang, J., Yao, J., Ma, N., Liang, Z., Huang, W., Huang, K., et al. (2020). JMJD3 and UTX determine fidelity and lineage specification of human neural progenitor cells. Nat Commun *11*, 382. https://doi.org/10.1038/s41467-019-14028-x.

Shimasaki, N., Jain, A., and Campana, D. (2020). NK cells for cancer immunotherapy. Nat. Rev. Drug Discov. *19*, 200–218. https://doi.org/10.1038/s41573-019-0052-1.

Urbańska, K., and Orzechowski, A. (2019). Unappreciated Role of LDHA and LDHB to Control Apoptosis and Autophagy in Tumor Cells. Int. J. Mol. Sci. *20*, 2085. https://doi.org/10.3390/ijms20092085.

Vivier, E., Rebuffet, L., Narni-Mancinelli, E., Cornen, S., Igarashi, R.Y., and Fantin, V.R. (2024). Natural killer cell therapies. Nature *626*, 727–736. https://doi.org/10.1038/s41586-023-06945-1.

Wang, J.X., Choi, S.Y.C., Niu, X., Kang, N., Xue, H., Killam, J., and Wang, Y. (2020). Lactic Acid and an Acidic Tumor Microenvironment suppress Anticancer Immunity. Int. J. Mol. Sci. *21*, 8363. https://doi.org/10.3390/ijms21218363.

Wang, X., Zhang, Y., Jin, Y., Dai, L., Yue, Y., Hu, J., Liu, X., Pang, K., Ye, S., Chen, Y., et al. (2025). An iPSC-derived CD19/BCMA CAR-NK therapy in a patient with systemic sclerosis. Cell *188*, 4225–4238.e12.

Wang, Z.H., Peng, W.B., Zhang, P., Yang, X.P., and Zhou, Q. (2021). Lactate in the tumour microenvironment: From immune modulation to therapy. EBioMedicine *73*, 103627. https://doi.org/10.1016/j.ebiom.2021.103627.

Wu, S.Y., Fu, T., Jiang, Y.Z., and Shao, Z.M. (2020). Natural killer cells in cancer biology and therapy. Mol. Cancer *19*, 120. https://doi.org/10.1186/s12943-020-01238-x.

Xue, D., Lu, S., Zhang, H., Zhang, L., Dai, Z., Kaufman, D.S., and Zhang, J. (2023). Induced pluripotent stem cell-derived engineered T cells, natural killer cells, macrophages, and dendritic cells in immunotherapy. Trends Biotechnol. *41*, 907–922. https://doi.org/10.1016/j.tibtech.2023.02.003.

Zhang, C., Kadu, S., Xiao, Y., Johnson, O., Kelly, A., O'Connor, R.S., Lai, M., Kong, H., Srivatsa, S., Tai, V., et al. (2023). Sequential Exposure to IL21 and IL15 During Human Natural Killer Cell Expansion Optimizes Yield and Function. Cancer Immunol. Res. *11*, 1524–1537. https://doi.org/10.1158/2326-6066.CIR-23-0151.

Zhang, T., Huang, K., Zhu, Y., Wang, T., Shan, Y., Long, B., Li, Y., Chen, Q., Wang, P., Zhao, S., et al. (2019). Vitamin C-dependent

lysine demethylase 6 (KDM6)-mediated demethylation promotes a chromatin state that supports the endothelial-to-hematopoietic transition. J. Biol. Chem. *294*, 13657–13670. https://doi.org/10.1074/jbc.RA119.009757.

Zheng, G., Shi, J., Li, Q., Jin, X., Fang, Y., Zhang, Z., Cao, Q., Zhu, L., and Shen, J. (2024). BAP1 inactivation promotes lactate production by leveraging the subcellular localization of LDHA in melanoma. Cell Death Discov. *10*, 483. https://doi.org/10.1038/s41420-024-02250-6.

Zhu, X., Xue, J., Jiang, H., and Xue, D. (2024). CAR-NK cells for gastrointestinal cancer immunotherapy: from bench to bedside. Mol. Cancer *23*, 237. https://doi.org/10.1186/s12943-024-02151-3.

Zhu, Y., Wang, T., Gu, J., Huang, K., Zhang, T., Zhang, Z., Liu, H., Tang, J., Mai, Y., Zhang, Y., et al. (2020). Characterization and generation of human definitive multipotent hematopoietic stem/progenitor cells. Cell Discov. *6*, 89. https://doi.org/10.1038/s41421-020-00213-6.

STAR★METHODS

KEY RESOURCES TABLE

REAGENT or RESOURCE	SOURCE	IDENTIFIER
**Antibodies**		
HRP-conjugated DYKDDDDK Tag Monoclonal antibody	proteintech	Cat#66008
GAPDH Antibody	Affinity	Cat#AF0911
Human TOMM20 Antibody	abcam	Cat#ab88207
Human CD45 APC Antibody	BD pharmingen	Cat#555485
Human CD107a APC Antibody	BD pharmingen	Cat#560664
Human CD56 BV421 Antibody	BD pharmingen	Cat#562751
Human CD43 FITC Antibody	BD pharmingen	Cat#560978
Human CD94 PE Antibody	BD pharmingen	Cat#555889
Human CD16 PE Antibody	BD pharmingen	Cat#561313
Human CD178 PE Antibody	BD pharmingen	Cat#564261
Human CD262 PE Antibody	BD pharmingen	Cat#565499
Human NKG2D PE Antibody	BD pharmingen	Cat#561815
Human NKP44 PE Antibody	BD pharmingen	Cat#558563
Human NKP46 PE Antibody	BD pharmingen	Cat#557991
**Bacterial and virus strains**		
PUC57-ROSA26-EF1a-Kozak-LDHB-Flag-puromycin	This paper	N/A
**Chemicals, peptides, and recombinant proteins**		
Ionomycin	Yeasen Biotechnology	50401ES03
Phorbol 12-myristate 13-acetate (PMA)	TargetMol	TQ0198
EIPA	GLPBIO	GC15893–5.1
Sodium lactate	Aladdin	S108838
L-Lactic Acid	Aladdin	L425989
**Critical commercial assays**		
ATP Assay Kit	Beyotime Biotechnology	S0026
NAD+/NADH Assay Kit with WST-8	Beyotime Biotechnology	S0175
L-Lactate Assay Kit with WST-8	Beyotime Biotechnology	S0208S
Intracellular pH Assay Kit with BCECF AM	Beyotime Biotechnology	C2250S
Human IFNr ELISA	Signosis	EA-0507
Enhanced BCA Protein Assay Kit	Beyotime Biotechnology	P0010S
**Experimental models: Cell lines**		
HiPSC: RS004	South China Stem Cell Bank	N/A

(*Continued on next page*)

Continued

REAGENT or RESOURCE	SOURCE	IDENTIFIER
U87-MG	South China Stem Cell Bank	N/A
SKOV3	South China Stem Cell Bank	N/A
**Experimental models: Organisms/strains**		
Mouse:NOD/ShiLtJGpt-Prkdcem26Cd52Il2rgem26Cd22/Gpt	GemPharmatech Co., Ltd.	NCG | Strain NO. T001475
**Oligonucleotides**		
LDHB-EcoR1-F	This paper	CGGAATTCCGATGGCAAACTCT TAAGGAAAAACTCATTG
LDHB-BamHI-R	This paper	CGACGGATCCTTACTTGTACAG CTCGTCCAT
Left-kozak-LDHB	This paper	CGTGAGGAATTCGCGACGCCGCC ACCATGGCAAACTCTTAAGGAAAA
Right-flag-LDHB	This paper	AGACCTGGATTACAAGGACGACGA TGACAAGTAAAGATCTATCGATAATC
GAPDH-qRT-F	This paper	GGCTGTTGTCATACTTCTCATGG
GAPDH-qRT-R	This paper	GGCTGTTGTCATACTTCTCATGG
FOS-qRT-F	This paper	CCGGGGATAGCCTCTCTTACT
FOS-qRT-R	This paper	CCAGGTCCGTGCAGAAGTC
JUNB-qRT-F	This paper	GCTCGGTTTCAGGAGTTTGTAGT
JUNB-qRT-R	This paper	CATCAGCCCGATGTACCAGC
LDHB-qRT-F	This paper	TGGTATGGCGTGTGCTATCAG
LDHB-qRT-R	This paper	TTGGCGGTCACAGAATAATCTTT
TOMM20-qRT-F	This paper	GGTACTGCATCTACTTCGACCG
TOMM20-qRT-R	This paper	TGGTCTACGCCCTTCTCATATTC
OCT4-qRT-F	This paper	GAACATGTGTAAGCTGCGGC
OCT4-qRT-R	This paper	GCCTCTCACTCGGTTCTCGA
**Recombinant DNA**		
PUC57-ROSA26-EF1a-Kozak-LDHB-Flag-puromycin	This paper	N/A
**Software and algorithms**		
ImageJ	National Institutes of Health	https://imagej.nih.gov/ij/
Seahorse Analytics	Agilent Technologies, Inc.	https://www.agilent.com/
GraphPad Prism	Dotmatics	https://www.graphpad.com/
FlowJo	BD Biosciences	https://www.flowjo.com/

EXPERIMENTAL MODEL AND STUDY PARTICIPANT DETAILS

Cell lines and cell culture

All hiPSCs and Knockin-LDHB-hiPSCs were cultured in mTeSR1 medium (Stem Cell Technologies) supplemented with 1% penicillin-streptomycin (Hyclone) on Matrigel-coated plates (1:200 dilution; BD Biosciences). The medium was replaced every day and the cells were digested and passaged with 0.5 mM EDTA (Sigma) every 3–4 days. All tumor cells, including

U87-luc and SKOV3-luc were cultured in Dulbecco's modified Eagle medium (DMEM) medium supplemented with 10% fetal bovine serum (FBS) and 1% penicillin–streptomycin. iNK were cultured in RPMI 1640 medium (Gibco) supplemented with 10% fetal bovine serum (FBS), 1% penicillin–streptomycin and 100IU IL2 (Sino Biological). All the cells were cultured in a humidified incubator with 5% CO2 at 37°C. hiPSCs were sourced from the South China Stem Cell Bank. All cell lines have no bacterial infections and tested negative for mycoplasma.

Mice

NOD-PrkdcscidIl2rgtm1/Bcgen (NCG) immunodeficient mice were purchased from GemPharmatech (Nanjing, China) and bred in the specific pathogen-free (SPF) animal facility at the Guangzhou Institutes of Biomedicine and Health (GIBH), Chinese Academy of Sciences. All mice were 6–8 weeks old at the start of experiments, female, and maintained under controlled environmental conditions (22 ± 2°C, 50 ± 10% relative humidity, 12-h light/dark cycle) with *ad libitum* access to sterile water and standard chow. Routine pathogen screening confirmed all mice were free of common murine pathogens including Mycoplasma spp.

All animal procedures were approved by the Institutional Animal Care and Use Committee of GIBH (Approval No. GIBH2025020) and conducted in strict accordance with the ARRIVE guidelines. Mice were randomly allocated into experimental groups using a random number generator prior to tumor inoculation, and investigators performing tumor measurement and data analysis were blinded to group allocation.

Hematopoietic differentiation with hESCs

The Hematopoietic differentiation was performed as previously described (Zhu et al., 2020). Briefly, hiPSCs were plated onto Growth Factor Reduced Matrigel (1:200 dilution; BD Biosciences)-coated plates at a proper initial density. The hematopoietic differentiation medium was changed every day, supplemented with corresponding cytokines or inhibitors, and floated hiPSCs-HSPCs were collected from day 8 for further assays. The cytokines or inhibitors added each day were as follows: day 0–2: 40 ng/mL BMP4 (Peprotech), 30 ng/mL ACTIVIN A (Sino Biological), 20 ng/mL bFGF (Sino Biological), 6 μM CHIR99021 (Selleck), 1 μM A8301 (Selleck); day 2–4: 40 ng/mL VEGF (Sino Biological) and 50 ng/mL bFGF; after day 4: 50 ng/mL bFGF, 50 ng/mL TPO (Sino Biological), 50 ng/mL IL6 (Sino Biological) and 50 ng/mL FLT3L (Peprotech).

Natural killer cells differentiation

hiPSCs-HSPCs were cocultured with OP9-hDL4 cells for about 14 days with differentiation system to differentiate into NKP cells, then NKP cells were cocultured with irradiated K562 cells for expansion to obtain iNK cells.

Gene targeting

To construct hPSCs with constitutive expression of LDHB in ROSA26 harbor locus, guide RNAs (gRNAs) for ROSA26 safe harbor locus were designed on our website and cloned into PUC57 (a vector that can express Cas9 protein and gRNA). Donor plasmid (PUC57-ROSA26-EF1a-Kozak-LDHB-Flag-puromycin) contained left and right homology arms of ROSA26 safe harbor locus. A total of 1×10^6 hPSCs were electroporated with 4μg donor plasmid and 2μg PUC57 plasmid, and cells were primarily selected by puromycin (0.5μg/mL). The positive individual clones were picked for further screening by PCR and Sanger sequencing.

METHOD DETAILS

CFU assay

Single cells of the certain cell number were suspended in the 100μL of Iscove's modified Dulbecco's medium (Gibico) supplemented with 2% FBS (Biological Industries) and then mixed with 1 mL of Methocult H4435 (Stem Cell Technologies) in 35-mm ultra-low attachment plates (Stem Cell Technologies). After the cell culture at 37°C and with 5% CO2 of 14 days, the CFUs were classified and counted according to their morphology.

RT-qPCR

Total RNA was isolated by the TRIzol (Sigma) following the manufacturer's instructions. Then, the RNA was reverse transcribed into cDNA by the HiScript III RT SuperMix for RT-qPCR (Vazyme). The RT-qPCR was performed on a CFX96 machine (Bio-Rad) with the SYBR Green Mix (Vazyme). PCR cycle conditions were at 95°C for 10 min for denaturation, 40 cycles of 95°C for 10 s, 60°C for 10 s and 72°C for 20s. All experiments were done in triplicate.

Flow cytometry

Cells were collected at indicated time and stained by multicolor antibody combinations in fluorescence-activated cell sorting (FACS) buffer (DPBS +2% FBS). After incubation at 4°C for 20–30 min, cells were washed and resuspended with FACS buffer for analysis by CytoFLEX (Beckman). For time-course expansion analysis, different samples of cells were processed uniformly and resuspended with 120 μL of FACS buffer in a tube. Subsequently, 30 μL from each tube was analyzed by CytoFLEX at a constant speed and total number for a tube calculated as 4-folds of the displayed number.

Cell-cycle and apoptosis analysis

Cell-cycle assay was performed by EdU and DAPI co-staining according to the manufacturer's instructions for the Click-iT EdU Pacific Blue flow cytometry assay kit (Invitrogen). A total of 5 × 105 differentiated cells were plated onto 24-well plates supplemented with 10 μM EdU or without EdU as a negative control for 4 h. Cells were collected by centrifugation and washed twice by DPBS then fixed with fixation buffer (BD Biosciences) at room temperature for 15 min. Then, cells were washed with DPBS and permeabilized in perm/wash buffer (BD Biosciences) at 4°C for 15 min. After that, cells were incubated with Alexa Fluor 647 azide at 37°C for 30 min and incubated with DAPI (1:200 dilution, Beyotime) at room temperature for 10–15 min. Finally, these cells were analyzed by CytoFLEX.

Propidium iodide (PI) staining was also performed to detect the cell cycle according to the manufacturer's instructions for the Cell Cycle and Apoptosis Analysis Kit (Beyotime).

Apoptosis assay was performed by Annexin V-FITC/PI Apoptosis Detection Kit (Vazyme) according to the manufacturer's instructions. We chose only Annexin V-APC to stain because the PI channel overlaps with the mCherry channel. Then the cells were analyzed by CytoFLEX.

Immunofluorescence

Cells were harvested by centrifugation and fixed by 4% paraformaldehyde (Jingxin Biology) at room temperature for 15 min. Next, the cells were washed and resuspended with DPBS at a density of 1×10^4/100 μL. Then, 10 μL of the cell mixture was added onto the glass slide and they were dried at room temperature. Then, cells were incubated with primary antibodies overnight at 4°C for 12–16 h followed by incubation with specific secondary antibodies at room temperature for 2 h. Finally, nuclei were stained with DAPI and analyzed with an LSM800 confocal microscope (Carl Zeiss).

Western Blot

WB assay was performed as previously described (Shan et al., 2020). In brief, total proteins were extracted using RIPA lysis buffer supplemented with protease and phosphatase inhibitors (Beyotime). Protein concentrations were determined via BCA (Beyotime) assay, and equal amounts of protein were loaded per lane. Samples were denatured by boiling in loading buffer, separated by SDS-PAGE, and subsequently transferred onto PVDF (Beyotime) membranes via wet transfer. The membranes were blocked with 5% BSA at room temperature for 1 h, followed by overnight incubation with primary antibodies at 4°C. After washing with TBST, membranes were incubated with HRP-conjugated secondary antibodies at room temperature for 1 h. Protein signals were visualized using enhanced chemiluminescence (ECL) and semi-quantitatively analyzed using ImageJ software. Detailed information for all antibodies used is provided in the key resources table. GAPDH was used as control. Band intensities were quantified using ImageJ software by measuring the integrated density of target proteins and GAPDH after subtracting local background values. Relative protein expression was calculated as the ratio of background-subtracted target intensity to GAPDH intensity, followed by normalization to the control group mean.

Measurement of intracellular pyruvate and lactate levels

Intracellular pyruvate and lactate levels were measured using commercially available fluorescence-based assay kits according to the manufacturer's instructions. Pyruvate concentrations were determined using the Amplex® Red Pyruvate Assay Kit (Catalog No. S0299S, Beyotime Biotechnology, China), and lactate levels were assessed with the Amplex® Red L-Lactate Assay Kit (Catalog No. S0027S, Beyotime Biotechnology, China).

Intracellular pH (pHi) measurement

Intracellular pH was measured using the BCECF-AM fluorescent probe according to the manufacturer's protocol (Beyotime Biotechnology, Cat# C2250S). Briefly, cells were harvested and resuspended in standard buffer containing 10 μM BCECF-AM at a concentration of 5×10^5 cells per tube, followed by incubation at 37°C for 10 min in the dark. After staining, cells were washed three times with PBS to remove excess extracellular dye. The fluorescence intensity of

BCECF-loaded cells was then analyzed using flow cytometry with excitation at 488 nm and emission detected through the FITC channel. The intracellular pH value was determined by interpolating the geometric mean fluorescence intensity against a standard calibration curve established using the kit's calibration buffers.

In vitro cytotoxicity assay

U87-MG and SKOV3 tumor cell expression Luciferase were used as targets in the luciferase-based killing assays. Expanded WT iNK and KI-LDHB-iNK were used as effector. The effector-to-target (E:T) ratio was serially titrated from 5:1 down to 0.2:1, Triplicate wells were established for each group. Bioluminescence was measured using an IVIS Spectrum (PerkinElmer). The luciferase signal (total flux) of the tumor-alone group was the control. Both tumor cell lines were obtained from the South China Stem Cell Bank.

Animal experiments

Animal experiment schemas are shown in detail in each relevant figure. Six-to eight-week-old female NCG mice were purchased from GemPharmatech and bred in the specific pathogen-free-grade animal care facility of the Guangzhou Institutes of Biomedicine and Health (GIBH), CAS. For the s.c. U87 tumor model, cells were s.c. injected into the flank in 100 mL PBS or 3 × 10 ˆ5 U87-luciferase cells in 100mL PBS at day 0; Three days later, WT-iNK or KI-LDHB-iNK in 100 mL PBS were injected at the same position of the tumor cells. Bioluminescent imaging was performed using an IVIS Spectrum (PerkinElmer), and analysis was performed using LivingImage (Caliper Life Sciences). For the survival experiment of iNK cells in solid tumors, U87 cells were s.c. into the groin of NCG mice, and 3 weeks later, when the tumor grew to about 0.5 cubic centimeters, WT-iNK or KI-LDHB-iNK cells were inoculated into the tumor by multi-point injection, then tumor was removed 3 days later, and the tumor was digested into single cells with type IV collagenase, and the number of NK cells was detected by flow cytometry. Mice were weighed and were subject to routine veterinary assessment for signs of overt illness. All of the animal experiments were performed according to the guidelines and were approved by the Institutional Animal Care and Use Committee at the Guangzhou Institutes of Biomedicine and Health (Approval No. GIBH2025020).

Statistical analysis

The data were presented as the mean ± SD from at least three independent experiments. For comparisons between two groups, the significance level was calculated by unpaired two-tailed Student's *t* test. For comparisons involving multiple groups and two independent variables, two-way analysis of variance (ANOVA) was used. A p value of <0.05 was considered statistically significant. Asterisks indicate significance as follows: *$p < 0.05$, **$p < 0.01$, and*$p < 0.001$. No samples were excluded for any analysis and no statistical method was used to pre-determine the sample size.

Stem Cell Reports, Volume *21*

Supplemental Information

Metabolic reprogramming via LDHB confers resistance to lactate-induced dysfunction and enhanced antitumor activity in hiPSC-Derived NK cells

Zhishuai Zhang, Yuchan Mai, Jun Tang, Jiaying Ning, Yanjiao Qin, Jiaming Gu, Zhaozong Cao, Jian Liu, Tiancheng Zhou, Junwei Wang, Jeane L. Pan, Jiaheng Chen, Yanling Zhu, and Guangjin Pan

supplemental 1

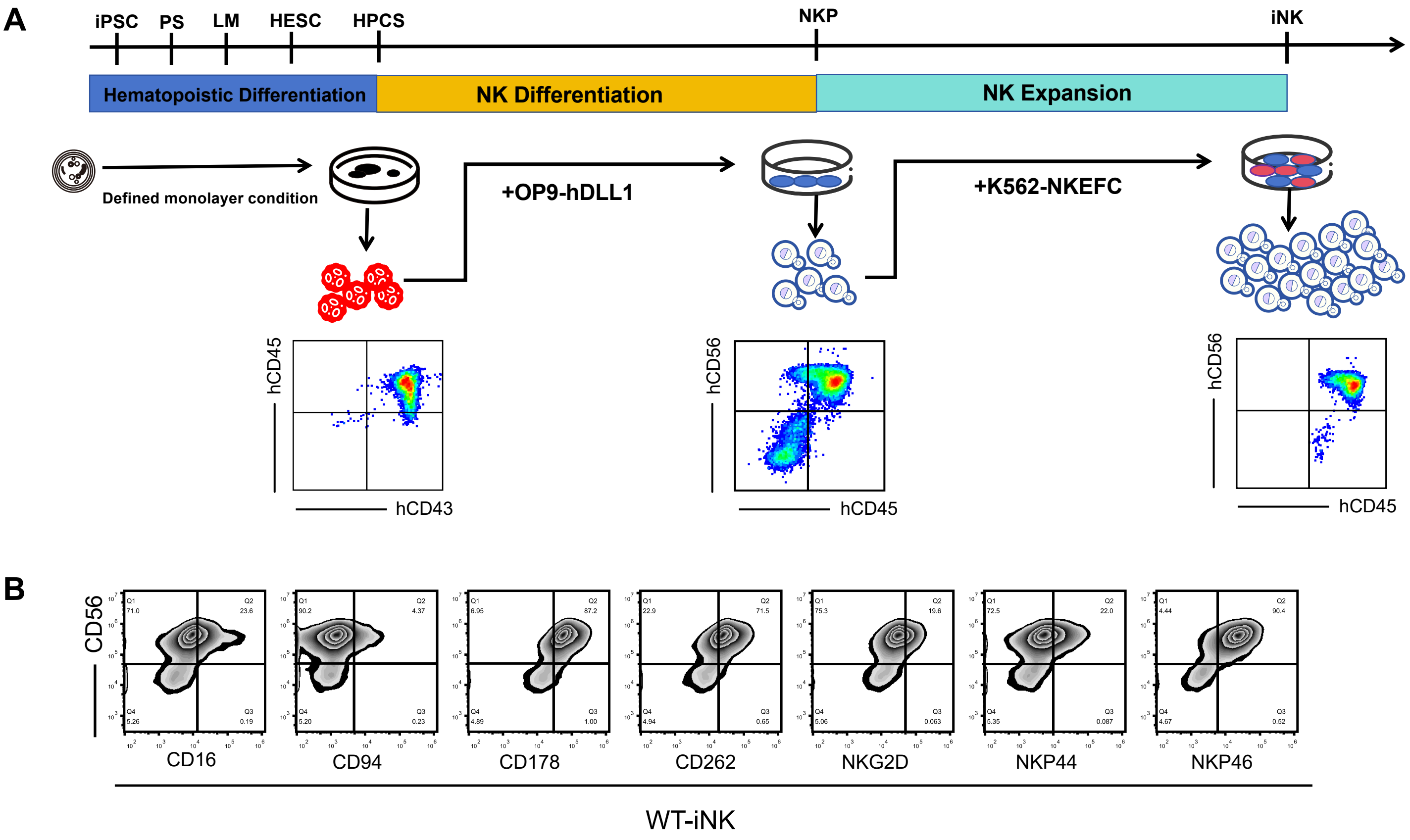

supplemental 2

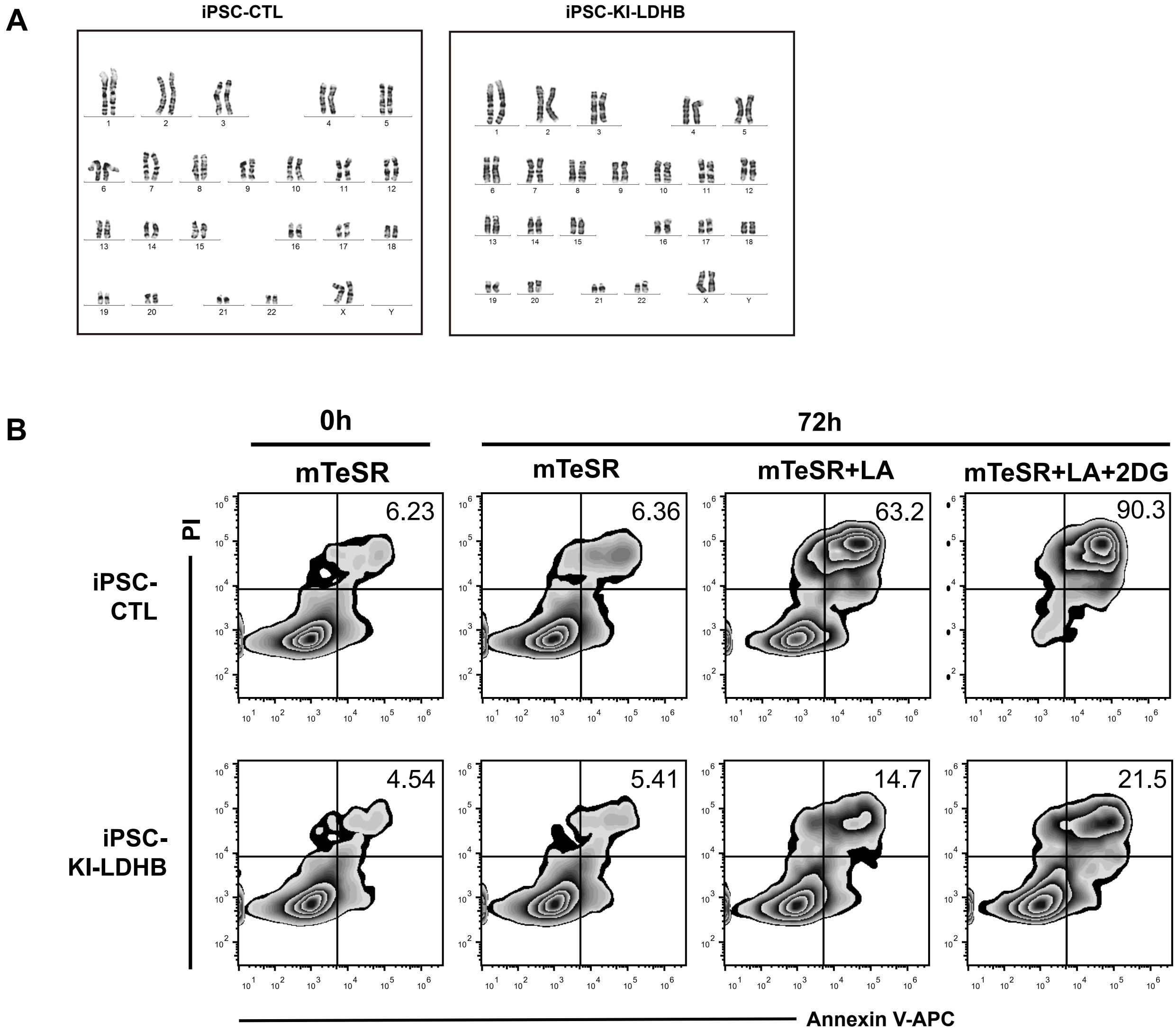

supplemental 3

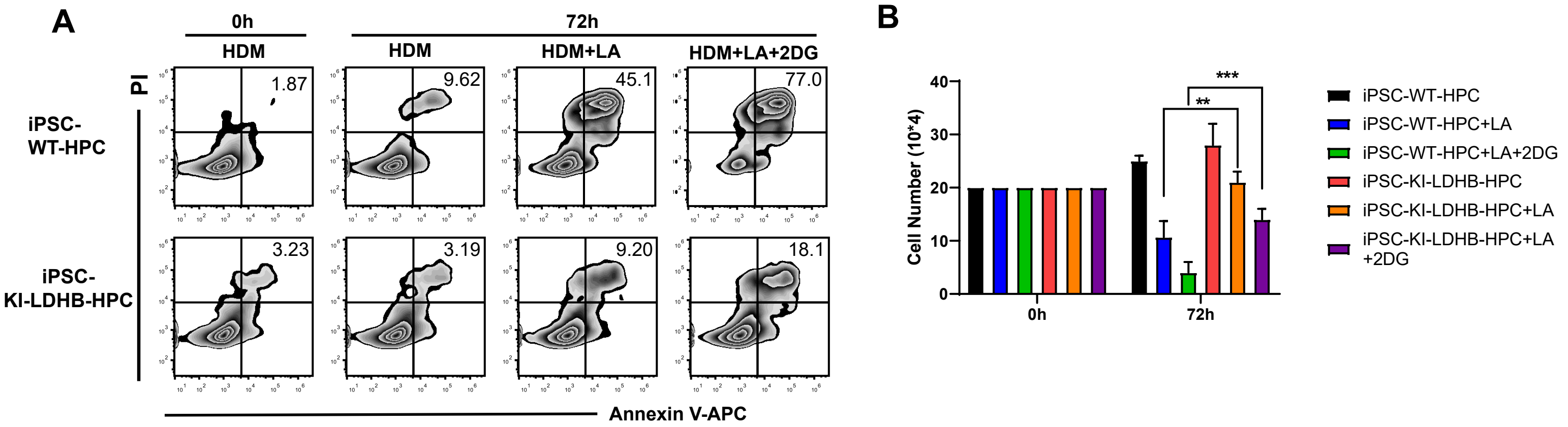

supplemental 4

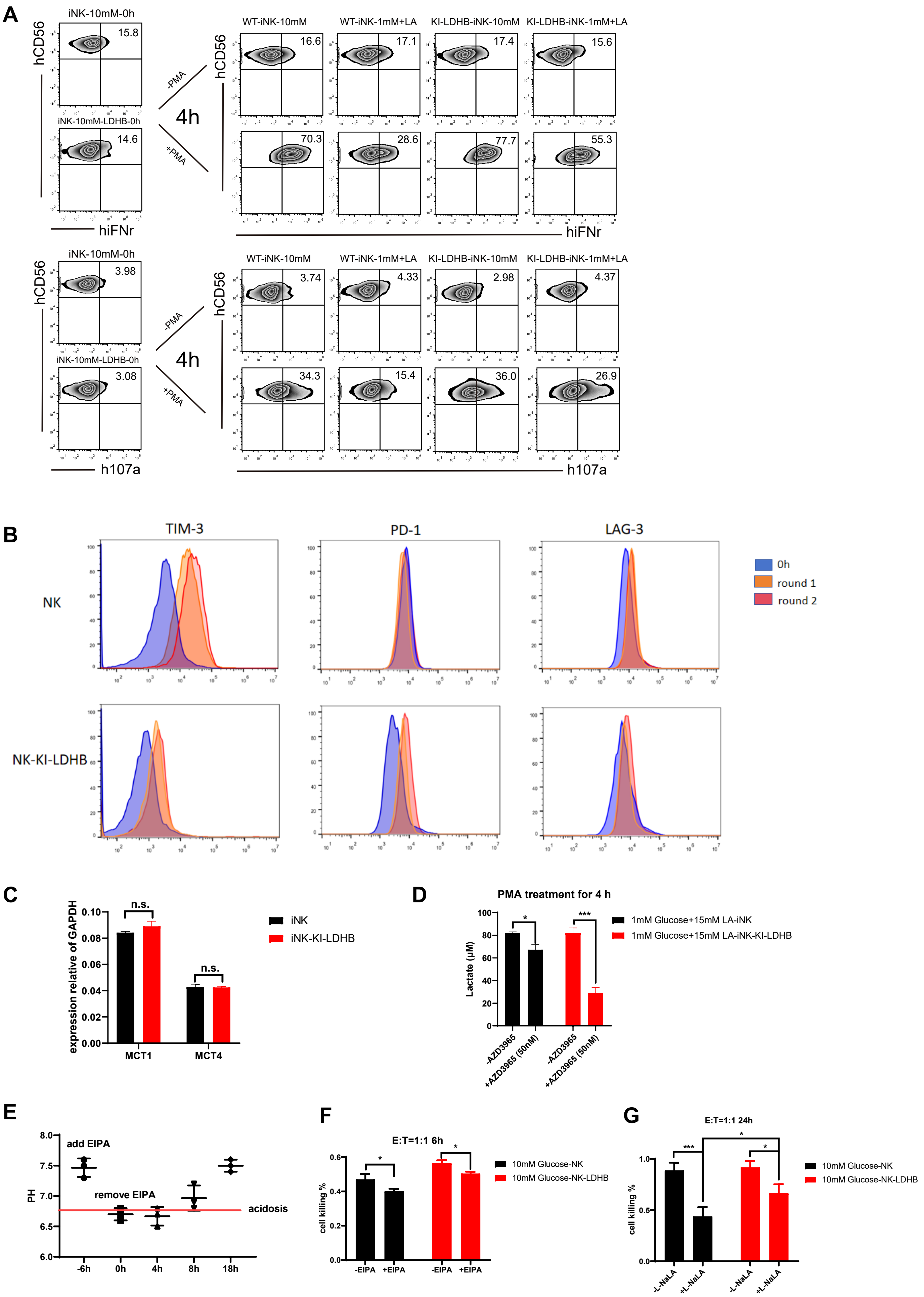

Supplemental 5

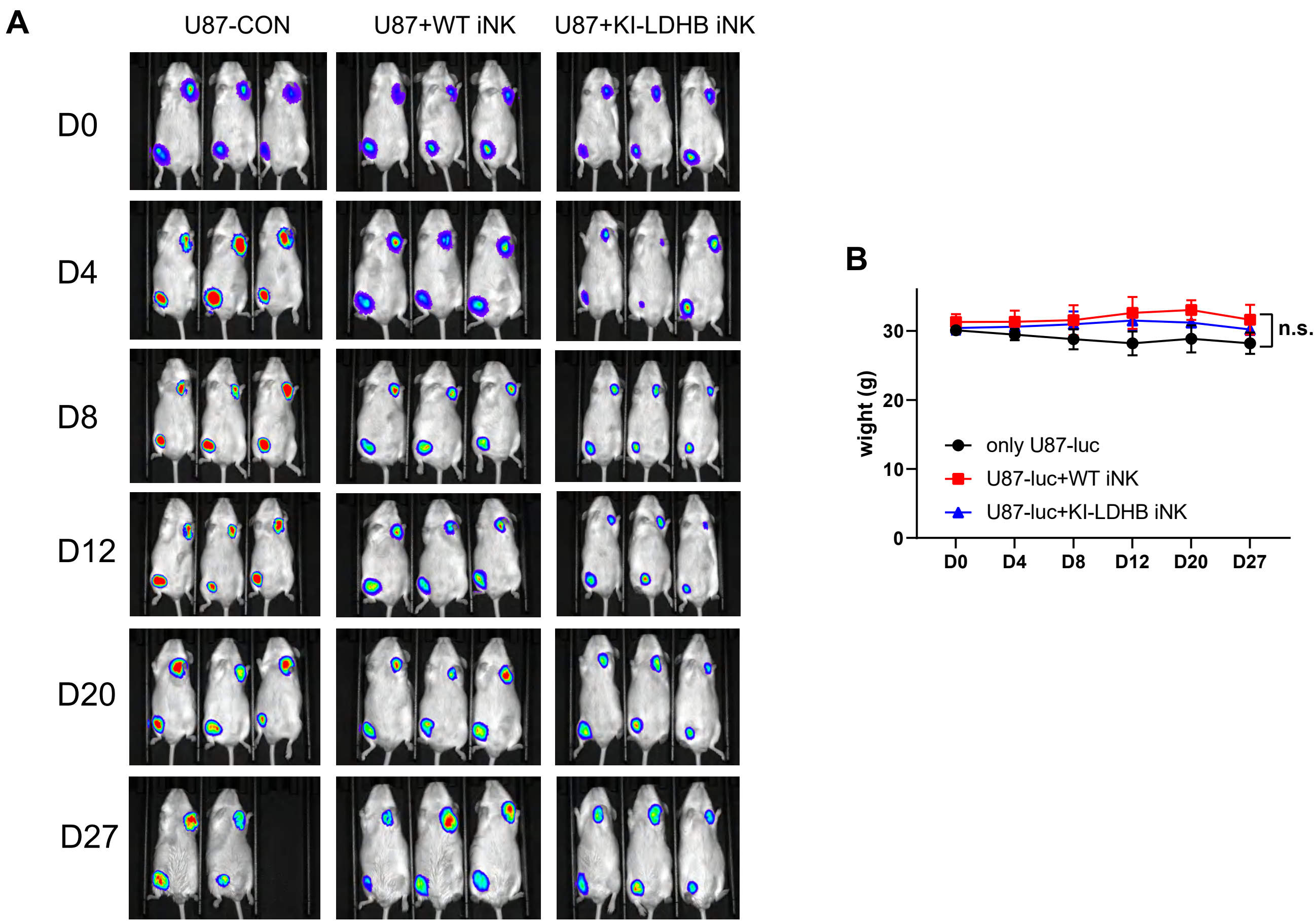

Supplemental Figure 1.

(A) Schematic of the differentiation protocol from hiPSCs to hematopoietic progenitor cells (iHPCs) to iNKs.

(B) Flow cytometry analysis characteristic NK cell surface markers on derived iNKs.

Supplemental Figure 2.

(A) Karyotype analysis of LDHB-knock-in iPSC clones.

(B) Flow cytometry analysis of apoptosis in WT and LDHB-hiPSCs after 72-hour culture in mTeSR, or mTeSR + lactate, or mTeSR + lactate + 2-DG.

Supplemental Figure 3.

(A) Flow cytometry analysis of apoptosis in WT and LDHB_iHSPCs after 72-hour culture in differentiation medium or medium + lactate, or medium + lactate + 2-DG.

(B) Quantification of cell numbers for the experiment in (A). $N=3$, Data are from three independent differentiation batches.

Supplemental Figure 4.

(A) Flow cytometry analysis of IFN-γ and Granzyme B secretion in WT and LDHB-iNKs, with or without lactate exposure, following PMA/ionomycin stimulation.

(B) Flow cytometry was used to detect the expression of exhaustion-related genes in iNK and iNK-KI-LDHB cells during multiple rounds of killing.

(C) Lactate concentration in iNK cells cultured in 1 mM glucose + 15 mM L-lactate medium , following 4 h PMA treatment. AZD3965 is a specific inhibitor of MCT-1, which can inhibit lactate influx.$N=3$, Data are from three independent differentiation batches. *$p < 0.05$, ***$p < 0.001$.

(D) Relative mRNA expression levels of MCT1 and MCT4 in iNK and iNK-KI-LDHB, normalized to GAPDH. $N=3$, Data are from three independent differentiation batches.

(E) Cells were treated with EIPA 40μM for 6 hours prior to the start of the experiment (time -6h). Upon removal of EIPA (time 0h), the intracellular pH gradually recovered but remained below the acidosis threshold (indicated by the red dashed line) throughout the observation period. *N=3*, Data are from three independent differentiation batches. Data are presented as mean ± SEM.

(F) Cytotoxic activity of iNK and iNK-KI-LDHB cells against U87 target cells at an effector-to-target (E:T) ratio of 1:1, measured after 6 hours of co-culture in EIPA medium. *N=3*, Data are from three independent differentiation batches. Data are mean ± SD . *$p < 0.05$, **$p < 0.01$.

(G) Cytotoxic activity of iNK and iNK-KI-LDHB cells against U87 target cells at an effector-to-target (E:T) ratio of 1:1, measured after 24 hours of co-culture in the presence or absence of 15 mM sodium L-lactate (L-A). *N=3*, Data are from three independent differentiation batches. Data are mean ± SD. *$p < 0.05$, **$p < 0.01$.

Supplemental Figure 5.

(A) Representative in vivo bioluminescence images of mice bearing U87-luc tumors treated with iNK or iNK-KI-LDHB.

(B) Body weight measurements of mice throughout the treatment period. *N=3*, with each set of data sourced from different mice.
